# Mortality attributable to hot and cold ambient temperatures in India: a nationally representative case-crossover study

**DOI:** 10.1371/journal.pmed.1002619

**Published:** 2018-07-24

**Authors:** Sze Hang Fu, Antonio Gasparrini, Peter S. Rodriguez, Prabhat Jha

**Affiliations:** 1 Centre for Global Health Research, St. Michael’s Hospital, Dalla Lana School of Public Health, University of Toronto, Toronto, Ontario, Canada; 2 Department of Public Health, Environments and Society, London School of Hygiene & Tropical Medicine, London, United Kingdom; 3 Centre for Statistical Methodology, London School of Hygiene & Tropical Medicine, London, United Kingdom; Africa Program, UNITED STATES

## Abstract

**Background:**

Most of the epidemiological studies that have examined the detrimental effects of ambient hot and cold temperatures on human health have been conducted in high-income countries. In India, the limited evidence on temperature and health risks has focused mostly on the effects of heat waves and has mostly been from small scale studies. Here, we quantify heat and cold effects on mortality in India using a nationally representative study of the causes of death and daily temperature data for 2001–2013.

**Methods and findings:**

We applied distributed-lag nonlinear models with case-crossover models to assess the effects of heat and cold on all medical causes of death for all ages from birth (*n* = 411,613) as well as on stroke (*n* = 19,753), ischaemic heart disease (IHD) (*n* = 40,003), and respiratory diseases (*n* = 23,595) among adults aged 30–69. We calculated the attributable risk fractions by mortality cause for extremely cold (0.4 to 13.8°C), moderately cold (13.8°C to cause-specific minimum mortality temperatures), moderately hot (cause-specific minimum mortality temperatures to 34.2°C), and extremely hot temperatures (34.2 to 39.7°C). We further calculated the temperature-attributable deaths using the United Nations’ death estimates for India in 2015. Mortality from all medical causes, stroke, and respiratory diseases showed excess risks at moderately cold temperature and hot temperature. For all examined causes, moderately cold temperature was estimated to have higher attributable risks (6.3% [95% empirical confidence interval (eCI) 1.1 to 11.1] for all medical deaths, 27.2% [11.4 to 40.2] for stroke, 9.7% [3.7 to 15.3] for IHD, and 6.5% [3.5 to 9.2] for respiratory diseases) than extremely cold, moderately hot, and extremely hot temperatures. In 2015, 197,000 (121,000 to 259,000) deaths from stroke, IHD, and respiratory diseases at ages 30–69 years were attributable to moderately cold temperature, which was 12- and 42-fold higher than totals from extremely cold and extremely hot temperature, respectively. The main limitation of this study was the coarse spatial resolution of the temperature data, which may mask microclimate effects.

**Conclusions:**

Public health interventions to mitigate temperature effects need to focus not only on extremely hot temperatures but also moderately cold temperatures. Future absolute totals of temperature-related deaths are likely to depend on the large absolute numbers of people exposed to both extremely hot and moderately cold temperatures. Similar large-scale and nationally representative studies are required in other low- and middle-income countries to better understand the impact of future temperature changes on cause-specific mortality.

## Abstract 2

Sze Hang Fu and colleagues reveal the effects of both cold and hot ambient temperatures on mortality rates in India from 2001-2013

## Author summary

**Why was this study done?:**

- Very few studies from low- and middle- income countries (LMICs) have examined daily hot and cold temperature effects on cause-specific mortality.
- This is, to our knowledge, the first study to estimate cause-specific deaths attributable to daily hot and cold temperatures in India using nationally representative mortality data spanning a 13-year period.

**What did the researchers do and find?:**

- We used a case-crossover method and distributed-lag nonlinear models (DLNM) to assess the nonlinear and delayed associations between temperature and mortality risk.
- We found substantial numbers of cause-specific deaths attributable to moderately cold temperature, which were approximately 12 times greater than deaths due to extremely cold temperature and 42 times greater than deaths due to extremely hot temperature.
- Our results also showed that moderately cold temperature was associated with the highest number of deaths from stroke at ages 30–69 years and from respiratory diseases at ages 70 years and above.

**What do these findings mean?:**

- Public health authorities should consider the detrimental effects of moderately cold and extremely hot temperatures in their mitigation strategies, particularly as the absolute population totals in India exposed to moderately cold and extremely hot temperatures have risen by about 270 and 10 million, respectively, in the last three decades.
- To provide reliable national estimates of temperature–mortality associations in other LMICs, large-scale and nationally representative mortality data are needed.

## Introduction

Most of the epidemiological studies that have examined the detrimental effects of ambient hot and cold temperatures on human health have been conducted in high-income countries [1,2]. A surprising conclusion has been that much of the excess mortality from temperature effects arises due to moderately cold temperatures [2]. Populations in low- and middle-income countries (LMICs) such as India are particularly vulnerable to adverse climate conditions due to having fewer physical adaptive measures such as air conditioning or heating, and less access to medical treatment for temperature-related health sequelae.

In India, the limited evidence on temperature and health risks has focused mostly on the effects of heat waves and has mostly been from small local studies in parts of India [3–6]. None of the focal studies that have examined cold effects are nationally representative [4,5,7]. Even fewer studies have examined the differences in temperature risk for cause-specific mortality [7]. Cardiovascular and respiratory diseases accounted for about 30% of total Indian adult deaths in 2010–2013 [8]. Robust and nationally representative estimates of the age- and cause-specific impact of both cold and hot temperatures are essential to mitigation strategies, particularly as the prediction of temperature and weather over a week to 10 days has improved substantially in the last two decades [9,10].

Here, we quantify the effects of heat and cold on all medical causes of death for all ages, as well as on stroke, ischaemic heart disease (IHD), and respiratory diseases among adults aged 30–69 in India. We use data from a nationally representative study of the causes of death and daily temperature data spanning a 13-year period.

## Methods

### Data sources

Our mortality data came from India’s Million Death Study (MDS) conducted in 2001–2013. The MDS is India's largest mortality survey undertaken by the Registrar General of India’s (RGI’s) Sample Registration System (SRS) [11]. It is national in scale and representative of both rural and urban settings. The RGI divides India into 1 million small areas of about 150–300 households based on the 10-year census [12]. The SRS randomly selects about 8,000 of these small areas and monitors all births and deaths in about 1.3 million households. Every six months, about 900 trained nonmedical surveyors visit these households and interview the relatives of people who have died in the past six months using a modified version of the 2016 WHO verbal autopsy form that includes a half-page local language narrative [13]. This information is converted to electronic records and randomly assigned to two of 404 trained physicians who classify the underlying causes of death using the WHO’s International Classification of Diseases (ICD)-10 [14]. Initial disagreements in coding undergo anonymous reconciliation by both physicians. Further disagreements are adjudicated by one of 40 senior physicians [12,15,16]. Address information from these death records permitted the geocoding of 565,282 deaths from 2001–2013, with urban deaths geocoded to postal code or town locations and rural deaths geocoded to post office or village locations. The SRS obtains oral consent from all participating households at the beginning of the 10-year SRS sampling frame. Households are free to withdraw, but, in reality, practically none do. Ethics approval for the MDS was obtained from the Post Graduate Institute of Medical Research, St. John’s Research Institute and St. Michael’s Hospital, Toronto, Ontario, Canada. Consent procedures have been published earlier [11,16,17].

We obtained daily gridded temperature data at 1 × 1° spatial resolution from the Indian Meteorological Department [18]. We extracted daily temperature values for each death by linking the deaths to the gridded data in time and space based on death dates and geographic locations. We excluded deaths without valid death dates (about 2% of all geocoded deaths, *n* = 9,557). We used daily mean temperature as the main exposure because it has been shown to predict temperature–mortality associations better than maximum and minimum temperatures [19].

### Statistical models

We utilized case-crossover methods to examine the transient effect of temperature exposure before the death occurred and compared that to exposure during control days [20,21]. Each death served as its own control, with control days matched to the same day of the week within the same month as when the death occurred [22]. This case-crossover model has the benefit of controlling for all time-invariant confounders, including individual characteristics (e.g. age, socioeconomic status) and local or regional effects [23]. Matching control days by day of the week and month avoids bias from systematic or slowly evolving temporal confounders such as day-of-week effects, seasonality, and time trends [22]. We applied distributed-lag nonlinear models (DLNM) with case-crossover models to assess the nonlinear and delayed associations between temperature and mortality risk [24]. With DLNM, we modeled the nonlinear temperature–mortality and lag–mortality associations using spline functions. A spline function consists of a sequence of polynomial segments joined by knots to form a single continuous curve [25]. The placement of knots controls the shape of the curve, and increasing the number of knots causes the curve to be more “wiggly.” We adopted similar knot placements and polynomial functions as used in a multicountry analysis [2] to facilitate comparisons across countries (see Section A in S1 Appendix for details). We modeled the lag–mortality associations using a lag period of up to 21 days to examine the delayed effects of cold and hot temperatures after the index day of exposure [26]. Currently, very few studies have fitted nonlinear and delayed effects of temperature on mortality in case-crossover models using individual deaths.

### All medical deaths analyses

**Fig 1:**
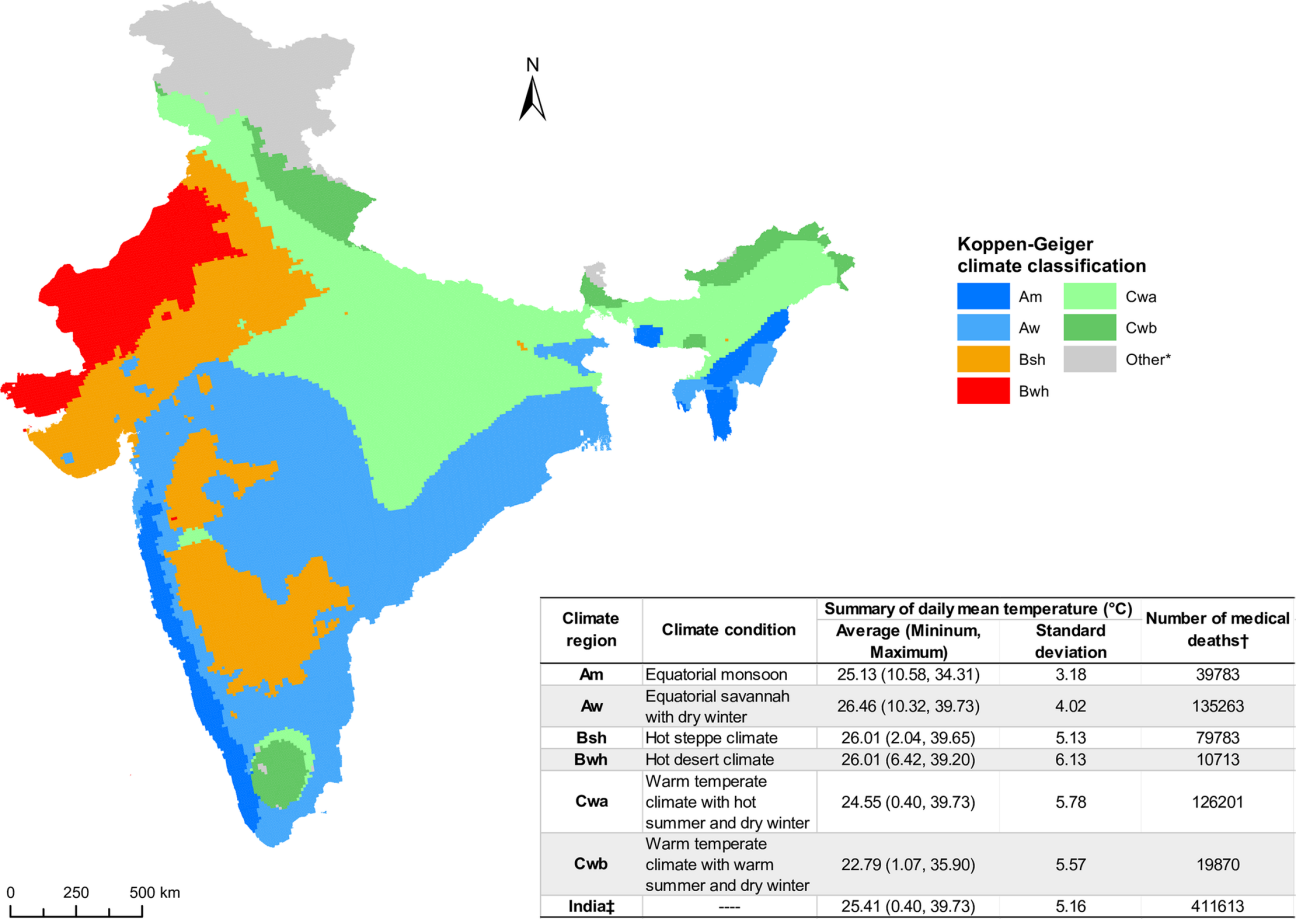
Major Köppen–Geiger climate classification and the associated summary statistics in India. **The six major climate regions are Am (equatorial monsoon), Aw (equatorial savannah with dry winter), Bsh (hot steppe climate), Bwh (hot desert climate), Cwa (warm temperate climate with hot summer and dry winter), and Cwb (warm temperate climate with warm summer and dry winter).** * "Other" included multiple climate regions in northern India. We excluded these regions from our analysis due to low death counts. † Medical deaths ICD-10 codes: A00-Q99, R04-06, R55, R84, R91, R96, X30-31. ‡ Summary statistics on daily mean temperature and number of deaths with six climate regions combined. ICD, International Classification of Diseases.

We applied a two-stage approach [2] to examine temperature associations with all known medical causes of deaths (excluding injury and ill-defined medical causes) at all ages and by age group (ages 0–29 years, 30–69 years, and 70 years and above) (see Table A in S1 Appendix for ICD-10 codes and death counts). The details of this flexible two-stage approach have been published elsewhere [27,28]. In the first stage, we used the Köppen–Geiger climate classification to aggregate deaths into six major Indian climate regions with notable variation in daily mean temperature (Fig 1). The Köppen–Geiger climate classification system is based on a large global dataset of long-term monthly climate data [29]. We ran separate case-crossover models for each climate region but excluded the northern regions with low death counts (*n* = 5,917) as these yielded unstable estimates. We reduced the temperature– and lag–mortality associations from the DLNM component of the case-crossover models to derive overall cumulative temperature–mortality associations by cumulating the risks during the lag period [30]. In the second stage, we combined the region-specific temperature–mortality associations to derive national pooled estimates using random effects meta-analysis, which accounted for differences in and precisions of temperature–mortality associations in region-specific estimates [31]. We tested residual heterogeneity between regions using the multivariate extension of the *I*^*2*^ statistic [31,32]. From each national temperature–mortality association, we obtained the minimum mortality temperature (MMT) and treated it as the centering value of the association. The MMT corresponded to the temperature with minimum mortality risk between the 1st and the 99th percentiles of the Indian temperature distribution for the SRS sampling units [2].

Similarly, we derived the pooled lag–mortality associations for cold and hot temperatures at the 1st and the 99th percentile thresholds, respectively, of the Indian temperature distribution using meta-analysis. We report temperature–mortality and lag–mortality associations with 95% confidence interval (CI).

### Cause-specific analyses

We performed analyses for stroke, IHD, respiratory diseases, malaria, and cancers for ages 30–69 years and ages 70 years and above (see Table A in S1 Appendix for ICD-10 codes and death counts) [15]. Due to smaller sample sizes, we combined these cause-specific deaths from the six climate regions to run separate case-crossover model for each mortality cause. We derived the overall cumulative temperature–mortality associations from the cause-specific models and centred these associations on the cause-specific MMTs. Malaria and cancers were chosen as control diseases for the temperature–mortality relationship. Earlier studies in high-income countries have shown that most cancer types [33] are not significantly associated with temperature. For malaria, parasites cannot easily develop inside their vector hosts under very cold and hot temperature conditions [34].

### Attributable risk fractions and number of deaths

Using the overall cumulative temperature–mortality associations, we calculated the temperature-attributable risk fractions by mortality cause for four temperature ranges: extremely cold, moderately cold, moderately hot, and extremely hot [2]. Cold and hot temperature ranges corresponded to below and above cause-specific MMTs, respectively; extreme and moderate temperature used cutoffs corresponding to the 2.5th and 97.5th temperature percentiles. These temperature ranges in degree Celsius were 0.4 to 13.8°C for extremely cold temperature, 13.8°C to cause-specific MMTs for moderately cold temperature, cause-specific MMTs to 34.2°C for moderately hot temperature, and 34.2 to 39.7°C for extremely hot temperature. We calculated empirical confidence intervals (eCIs) for the attributable risk fractions at 95% using Monte Carlo simulations with individual deaths as the resampling unit, assuming a multivariate normal distribution for the overall cumulative temperature–mortality associations [35]. These attributable risk fractions can be interpreted as an individual’s probability of dying, which, when summed, provide the attributable risk fraction at the sampled population level. We further calculated the temperature-attributable number of deaths by applying the attributable risk fractions and cause-specific proportionate mortality for 2001–2013 to the United Nations’ age-specific death estimates for India in 2015 [36] (see Section B in S1 Appendix for formula).

### Sensitivity analyses

We ran single-stage models by combining medical causes of deaths from all six climate regions by age group. We also assessed model sensitivity to different knot placements for the temperature–mortality associations from both two-stage meta-analyses for medical deaths and cause-specific models (see Section A in S1 Appendix for detail).

We performed all statistical analyses in R (3.3.3, main packages used were “dlnm,” “survival,” “mvmeta,” and “attrdl”). Sample R codes can be obtained from the first author.

We do not have a prospective analysis plan for this study as it is hypothesis driven.

## Results

**Fig 2:**
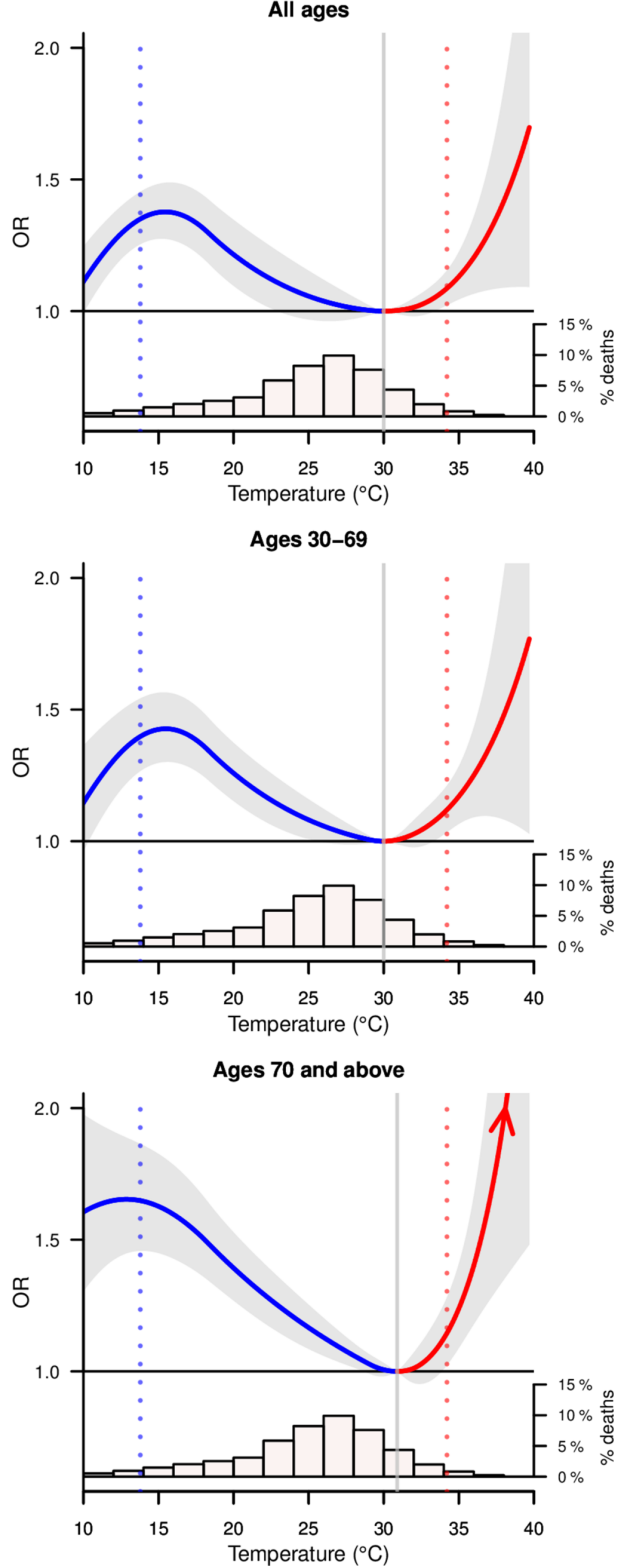
Overall cumulative temperature–mortality associations for medical deaths by age group. Solid curves in blue (estimates below MMTs) and red (estimates above MMTs) show pooled estimates of the temperature–mortality associations with 95% CIs (shaded grey); red arrow indicates increasing ORs for hot temperatures. Histograms show the proportion of deaths by daily mean temperature of the death date. Vertical grey solid lines represent the MMTs. Vertical blue and red dotted lines indicate the 2.5th and 97.5th percentiles of Indian temperature distribution. Graphs are restricted to 10°C–40°C due to wide CIs for extremely cold temperature. CI, confidence interval; MMT, minimum mortality temperature; OR, odds ratio.

The MDS recorded a total of 591,121 deaths from 2001–2013 from all causes, of which we geocoded 546,360 with complete death information and assigned them to the six climate regions. The main analyses focused on 411,613 medical deaths at all ages from 2001–2013 from six climate regions (Fig 1). The first-stage regional estimates showed distinctive temperature–mortality association by climate region for medical deaths (Fig A-D in S1 Appendix). Regions with lower death counts (e.g., Am, Bwh, Cwb) had higher uncertainty in their estimates. For the pooled estimates, most of the relevant medical conditions affected by temperature occurred above age 30 years. Mortality from all medical causes at ages 30–69 years showed excess risks at moderately cold temperature and hot temperature (Fig 2, middle panel). The lowest mortality risk was at 30°C and rose sharply at hot temperatures, averaging about a 9% excess risk with every degree temperature increase from 35°C to 40°C. Similarly, the all-medical mortality risk rose with lower temperature, averaging about a 3% excess risk with every degree drop in temperature from 30°C to 16°C, at which point the risks inflected and decreased. This decreasing risk at extremely cold temperature might be an artifact of the spline parameterization and due to the small number of deaths occurring on extremely cold days (Fig P in S1 Appendix). Mortality risks were similar for all ages (Fig 2, top panel) and ages 70 years and above (bottom panel), but with significant and steeper risks for hot temperature for ages 70 years and above.

**Fig 3:**
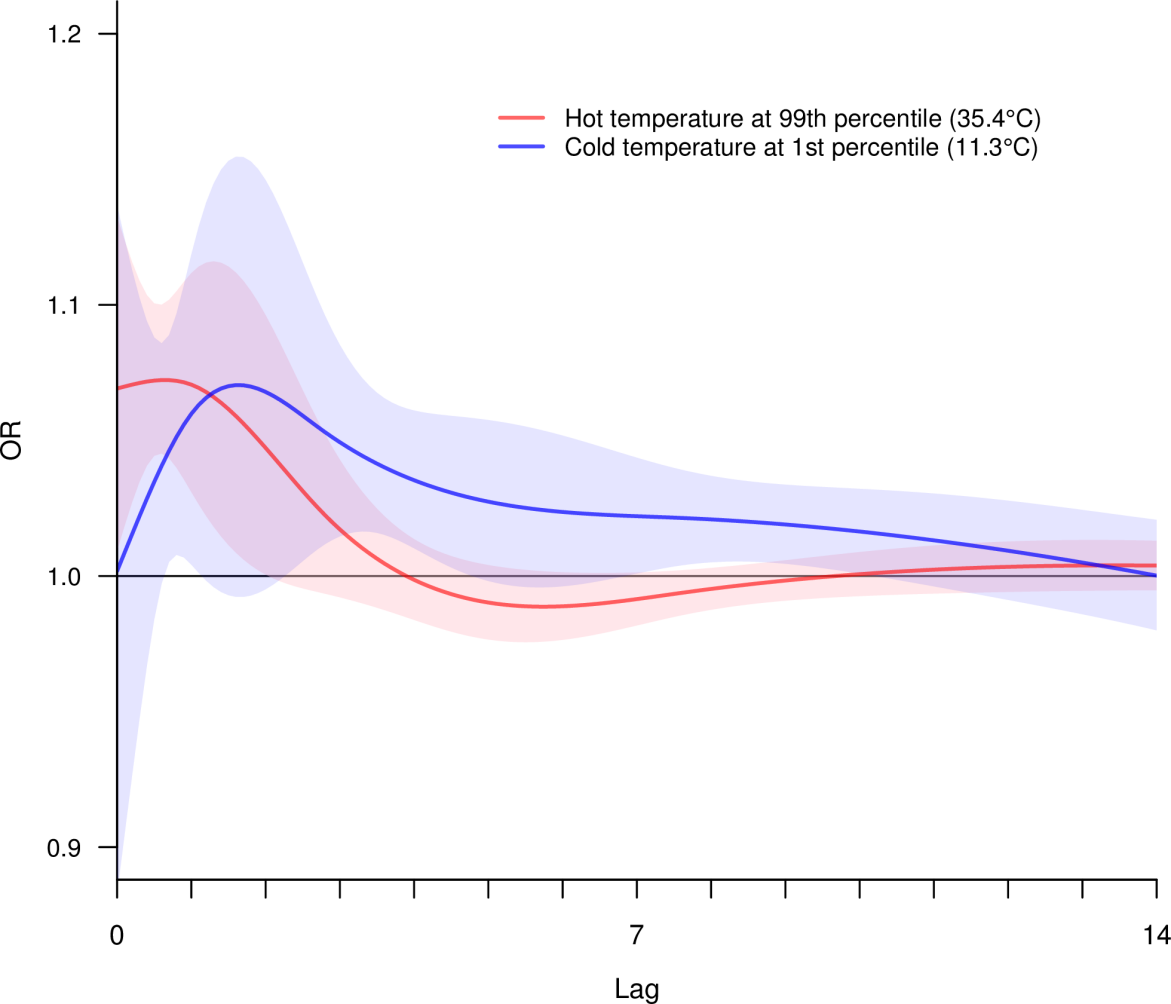
Lag–mortality associations from pooled estimates for medical deaths at ages 30–69 years. Solid curves represent lag–mortality associations, and shadings represent 95% CIs (red: hot temperature at 99th percentile; blue: cold temperature at 1st percentile). Graph is restricted to 0–14 lag days due to nonsignificant or negative ORs at 15–21 lag days. CI, confidence interval; OR, odds ratio.

The time lag analyses of temperature and mortality from all medical causes at ages 30–69 years showed hot temperature to yield an early peak in mortality risk within 0–1 days but no significant excess risks beyond 4 days (Fig 3). At 4–9 days, hot temperature showed negative risks, albeit with a lack of statistical significance. By contrast, the peak effects of cold temperature occurred on about day 2, and the excess risks persisted for about 14 days. These relationships were notably stronger for ages 70 years and above (Fig E in S1 Appendix, bottom panel) but were also noted at all ages (top panel).

**Fig 4:**
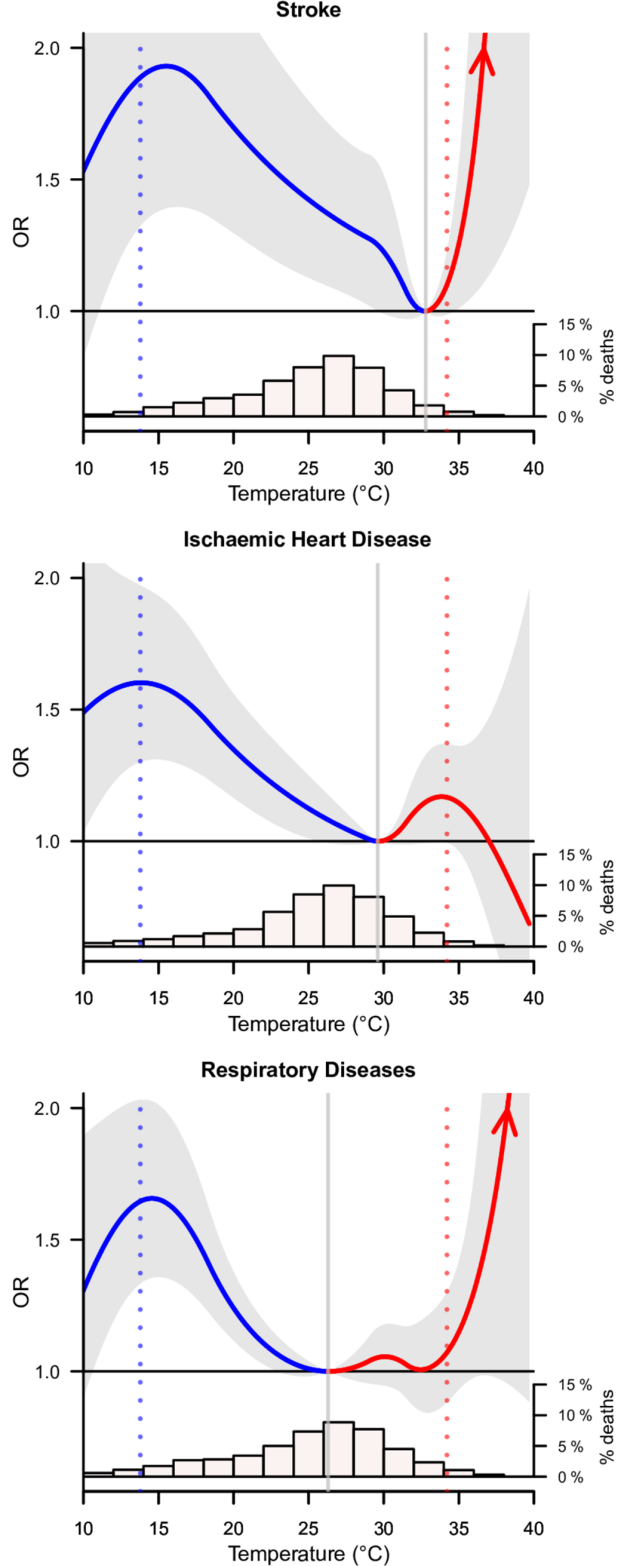
Overall cumulative temperature–mortality associations for specific mortality causes at ages 30–69 years. Solid curves in blue (estimates below MMTs) and red (estimates above MMTs) show the temperature–mortality associations with 95% CIs (shaded grey); red arrows indicate increasing ORs for hot temperatures. Histograms show the proportion of deaths by daily mean temperature of the death date. Vertical grey solid lines represent the model-specific MMTs. Vertical blue and red dotted lines indicate the 2.5th and 97.5th percentiles of Indian temperature distribution. Graphs are restricted to 10°C–40°C due to wide CIs for extremely cold temperature. CI, confidence interval; MMT, minimum mortality temperature; OR, odds ratio.

The relationship between temperature and cause-specific deaths, namely stroke, IHD, and respiratory conditions, are much more reliably studied at ages 30–69 years, as these deaths had far fewer ill-defined causes of death (about 8%) than at ages 70 years and above (about 28%). Moreover, these conditions were far less common below age 30 years. The overall distributions of mortality at ages 30–69 years from all medical causes and from these three conditions were similar to the overall daily mean temperature distribution in India (Fig P in S1 Appendix), with most deaths occurring not at extremely hot or cold temperatures. However, respiratory and stroke deaths were slightly more likely to occur at lower temperatures than were all medical deaths. At ages 30–69 years, hot temperatures showed steep and similar increases in mortality risk from stroke and respiratory diseases, but only the association with stroke was statistically significant (Fig 4, top panel; about 51% excess risk for every degree increase from 35°C to 40°C). Cold temperatures showed significant relationships for stroke, IHD, and respiratory conditions, with that for stroke being notably steeper (about 4% excess risk for every degree decrease from 30°C to 16°C). The relationship with extremely cold temperature was slightly stronger for IHD among the subset of these deaths at ages 30–69 years for people whose families reported a previous medical history of past vascular disease than those who did not (Fig G in S1 Appendix).

Malaria deaths at ages 30–69 years showed negative relationships at extremely cold and extremely hot temperatures, but not significantly so (Fig H in S1 Appendix, top panel). Significant but modest increases in risk of malaria deaths were observed at 14°C–20°C, consistent with the temperature range at which malaria parasites survive and reproduce [34]. Cancer deaths did not show any significant relationship with temperature (Fig H in S1 Appendix, bottom panel).

The *I*^*2*^ statistics for between-region heterogeneity of temperature–mortality pooled estimates for all medical causes were 39.5% (*p* = 0.0211) for all ages, 11.1% (*p* = 0.3021) for ages 0–29 years, 1.0% (*p* = 0.5453) for ages 30–69 years, and 35.2% (*p* = 0.0405) for ages 70 years and above. Thus, deaths at ages 70 years and above were the main contributors to the between-region heterogeneity in the pooled estimates for all ages. In sensitivity analyses, single-stage models produced almost identical results as the pooled estimates (Fig I in S1 Appendix). The use of different knot placements produced broadly similar results for the pooled estimates (Fig J-L in S1 Appendix) and the cause-specific estimates (Fig M-O in S1 Appendix).

For medical deaths at all ages, cold temperature (6.8%, 95% eCI 1.4 to 11.6) was associated with higher attributable risk than hot temperature (0.5%, 0.1 to 0.9). When separated by moderate and extreme temperature ranges, moderately cold temperature was associated with a higher attributable risk (6.3%, 1.1 to 11.1) than extremely cold, moderately hot, and extremely hot temperatures, each of which were less than 0.6% (Table 1). Medical deaths at ages 30–69 years and ages 70 years and above showed similar patterns, with much higher risks attributed to moderately cold temperature than to extremely cold, moderately hot, and extremely hot temperatures. For cause-specific deaths at ages 30–69 years, moderately cold temperature was associated with attributable risks of 27.2% (95% eCI 11.4 to 40.2) for stroke, 9.7% (3.7 to 15.3) for IHD, and 6.5% (3.5 to 9.2) for respiratory diseases; other temperature ranges showed attributable risks ranging from 0.1% to 1.2%. For cause-specific deaths at ages 70 years and above, moderately cold temperature was associated with attributable risks of 23.0% (95% eCI 5.8 to 36.6) for stroke, 16.3% (-9.1 to 35.8) for IHD, and 18.1% (11.2 to 24.3) for respiratory diseases; other temperature ranges had attributable risks ranging from 0.0% to 2.5%. Attributable risks were generally higher in ages 70 years and above than ages 30–69 years for both medical and cause-specific deaths.

**Table 1:** Mortality risks attributable to hot and cold ambient temperatures in India.

Underlying cause of death and age group	MMT	Extremely cold, 0.4°C to 13.8°C (95% eCI)	Moderately cold, 13.8°C to model-specific MMT (95% eCI)	Moderately hot, model-specific MMT to 34.2° C (95% eCI)	Extremely hot, 34.2°C to 39.7°C (95% eCI)
**All medical**					
All ages	30.0	0.5% (0.2 to 0.7)*	6.3% (1.1 to 11.1)*	0.2% (−0.1 to 0.5)	0.3% (0.1 to 0.4)*
Ages 0–29	24.9	0.0% (−0.8 to 0.5)	3.0% (1.9 to 4.1)*	3.1% (−2.7 to 7.8)	0.4% (−0.1 to 0.7)
Ages 30–69	30.0	0.5% (0.2 to 0.9)*	7.6% (3.4 to 11.5)*	0.3% (−0.1 to 0.8)	0.3% (0.1 to 0.5)*
Ages 70+	30.8	1.3% (0.9 to 1.6)*	12.5% (8.2 to 16.5) *	0.2% (−0.2 to 0.6)	0.4% (0.2 to 0.6)*
**Cause-specific, ages 30–69**					
Stroke	32.8	0.9% (−0.1 to 1.5)	27.2% (11.4 to 40.2)*	0.1% (−0.0 to 0.1)	0.4% (0.0 to 0.7)*
IHD	29.6	1.2% (0.4 to 1.8)*	9.7% (3.7 to 15.3)*	1.0% (−0.1 to 2.0)	0.2% (−0.2 to 0.5)
Respiratory diseases	26.3	1.1% (−0.1 to 1.9)	6.5% (3.5 to 9.2)*	1.1% (−2.0 to 3.9)	0.4% (−0.1 to 0.8)
**Proportion of days within temperature range for India in 2001–2013**		2.9%	80.6%	14.2%	2.3%

Notes: We used model-specific MMTs (ranged from 24.9°C to 32.8°C) as cutoffs for cold and hot temperatures and the 2.5th and 97.5th temperature percentiles as cutoffs for extreme and moderate temperatures.

* Estimates were statistically significant at 95% eCIs.

Abbreviations: eCI, empirical confidence interval; IHD, ischaemic heart disease; MMT, minimum mortality temperature.

Table 2 presents the estimated temperature-attributable number of deaths in 2015 for overall mortality and for ages 30–69 by cause of death. Moderately cold temperature was associated with the highest number of deaths for both medical and specific mortality causes. At all ages, 584,000 (95% eCI 385,000 to 763,000) medical deaths were attributable to moderately cold temperature. Among specific mortality causes, stroke contributed to the highest numbers of deaths (95,000, 95% eCI 40,000 to 140,000) at ages 30–69 years and respiratory diseases contributed most (101,000, 63,000 to 136,000) at ages 70 years and above due to deaths attributable to moderately cold temperature. Cold temperature ranges were associated with higher number of attributable deaths at ages 70 years and above than at ages 30–69 years. Total cause-specific deaths at ages 30–69 years attributable to moderately cold temperature were 197,000 (95% eCI 121,000 to 259,000), which was approximately 12 times greater than deaths due to extremely cold temperature and 42 times greater than deaths due to extremely hot temperature.

**Table 2:** Deaths (in thousands) attributable to hot and cold ambient temperatures in India in 2015.

Underlying cause of death and age group	Extremely cold, 0.4°C to 13.8°C (95% eCI†)	Moderately cold, 13.8°C to model-specific MMT (95% eCI†)	Moderately hot, model-specific MMT to 34.2°C (95% eCI†)	Extremely hot, 34.2°C to 39.7°C (95% eCI†)
**All medical**				
Ages 0–29	0.0 (−11.6 to 7.6)	47.0 (29.1 to 63.9)*	47.3 (−42.4 to 121)	5.5 (−1.8 to 11.5)
Ages 30–69	18.2 (5.1 to 28.6)*	254.1 (113.8 to 383.0)*	11.2 (−4.5 to 25.7)	10.4 (4.4 to 15.6)*
Ages 70+	29.6 (20.4 to 36.9)*	283.1 (186.3 to 373.9)*	5.1 (−4.8 to 14.4)	9.2 (4.5 to 13.2)*
Total†	47.8 (16.8 to 70.6)*	584.3 (384.8 to 763.4)*	63.6 (−13.6 to 129.0)	25.1 (15.4 to 33.2)*
**Cause-specific, ages 30–69**				
Stroke	3.1 (−0.4 to 5.2)	94.8 (39.8 to 140.0)*	0.2 (−0.1 to 0.5)	1.4 (0.0 to 2.6)*
IHD	8.9 (2.9 to 13.5)*	71.3 (27.2 to 112.4)*	7.4 (−0.8 to 14.9)	1.3 (−1.2 to 3.5)
Respiratory diseases	5.0 (−0.6 to 9.1)	30.6 (16.6 to 43.1)*	5.0 (−9.3 to 18.4)	1.9 (−0.5 to 3.9)
Total†	17.1 (8.0 to 23.1)*	196.7 (121.1 to 259.4)*	12.6 (−2.2 to 25.9)	4.7 (1.9 to 6.7)*

Notes: We used model-specific MMTs (ranged from 24.9°C to 32.8°C) as cutoffs for cold and hot temperatures and the 2.5th and 97.5th temperature percentiles as cutoffs for extreme and moderate temperatures.

*Estimates were statistically significant at 95% eCIs.

†We derived the eCIs for total temperature-attributable deaths for medical and specific mortality causes as follows: 1) For each repetition from Monte Carlo simulations, we summed the attributable deaths across subgroup. 2) We then obtained the 95% eCI (corresponded to the 2.5th and 97.5th percentiles) from the distribution of simulation repetitions with summed values.

**Abbreviations:** eCI, empirical confidence interval; IHD, ischaemic heart disease; MMT, minimum mortality temperature.

## Discussion

We demonstrate that cold temperatures contributed to higher attributable risks of mortality than hot temperatures in India during 2001–2013, consistent with previous findings based on nonlinear temperature–mortality associations drawn from mostly high-income countries [2]. These previous findings reported an overall attributable risk of 7.29% (eCI 7.02 to 7.49) for cold temperature and 0.42% (0.39 to 0.44) for hot temperature, which are similar to our results for all ages (Table C in S1 Appendix). Among countries, our results are most comparable to those of Australia.

We document a substantially greater number of deaths attributable to moderately cold temperature than to extremely cold and hot temperatures. The greater attributable risk of moderately cold temperature was partly due to the higher proportion of moderately cold days than extremely cold and hot days in India (Fig P in S1 Appendix). Also, small numbers of deaths occurring on extremely cold and hot days resulted in high uncertainty in the estimates for extreme temperatures. Previous small and focal studies in rural western India and Delhi found stronger heat effects on mortality than cold effects [5,7]. These studies did not have the benefits of our case-crossover methodology (e.g., controlling for time-invariant confounders) and relied on log-linear functions to model the temperature–mortality associations [5].

We identified differences in the temperature–mortality associations between stroke, IHD, and respiratory diseases. Moderately cold temperature had higher attributable risks for stroke than for IHD and respiratory diseases at both ages 30–69 years and ages 70 years and above. In absolute numbers, stroke and respiratory diseases accounted for the highest number of deaths at ages 30–69 years and ages 70 years and above, respectively, due to moderately cold temperature.

The significant cold effects on stroke, IHD, and respiratory diseases could be explained by physiological pathways between cold temperature and these diseases. Cardiovascular stress may be triggered by cold via changes in blood composition, which lead to increases in blood platelets, red cells, and viscosity; these changes facilitate vasoconstriction and blood clotting [37]. The respiratory system responds to cold by increasing bronchoconstriction, airway congestion, secretion, and decreasing mucociliary clearance; these changes may play a role in the symptoms of respiratory diseases, including asthma and chronic obstructive pulmonary diseases [38].

Overall, the elderly population had higher attributable risks from both hot and cold temperatures in our study [39]. Physiologically, the higher level of initial arterial diseases might cause elderly people to be more susceptible to changes in blood composition via changes in body temperature [37]. Also, the tolerance of and responsiveness to thermal extremes are more limited in the elderly [40].

The lag–mortality associations showed that hot temperature had a more immediate effect and that cold temperature had delayed and sustained effects, which are commonly observed in other studies [26,41]. Our results also showed that at hot temperature, a short duration of elevated mortality risks was followed by protective effects. This phenomenon is known as mortality displacement, which is thought to arise when mortality is brought forward in time for vulnerable individuals during stress events [26]. The elderly had stronger mortality displacement since they are likely to be more vulnerable than younger age groups.

In South Asia, average annual temperatures could rise by more than 2°C by the mid-21st century compared to the average in the 20th century [42]. This would be expected to increase heat-related deaths and reduce cold-related deaths [43]. However, nonclimatic factors also determine the ability of the population to adapt to climate change, including socioeconomic status [44,45] and the capability of the health system to treat medical conditions affected by temperature. Indeed, we note that over the last three decades, the proportion of moderately hot and extremely hot days has increased, and the proportion of extremely cold and moderately cold days has decreased in India (Table 3). However, the absolute number of people exposed to moderately cold temperatures is largest and has risen by about 270 million since 1981. The relative risks for extremely hot temperatures were more extreme than for moderately cold temperatures. Future absolute totals of temperature-related deaths are likely to depend on the large absolute numbers of people exposed to both extremely hot and moderately cold temperatures. Further studies using data from LMICs to relate mortality and projected climatic conditions are required to validate future changes in heat- and cold-related deaths and to investigate the extent of human acclimatization to the warming climate.

**Table 3:** Proportion of days within and population exposed to temperature ranges in India.

**Period**	**A) Proportion of days within temperature range in India**			
	**Extremely cold (−0.3°C to 13.8°C)**	**Moderately cold (13.8°C to 30.0°C)**	**Moderately hot (30.0°C to 34.2˚C)**	**Extremely hot (34.2°C to 40.2˚C)**
1981–1990	2.94%	82.00%	13.08%	1.98%
1991–2000	2.96%	81.90%	13.01%	2.13%
2001–2010	2.74%	80.46%	14.50%	2.31%
Relative change	−7%	−2%	+11%	+17%
**Period**	**B) Population (in millions) exposed to temperature range in India**			
1981–1990	20.1	560.3	89.4	13.5
1991–2000	25.1	693.2	110.1	18.1
2001–2010	28.2	827.7	149.1	23.7
Relative change	+40%	+48%	+67%	+76%
Absolute increase	8.1	267.3	59.8	10.2

Notes: A) Proportions were for daily mean temperatures from temperature grids that belonged to the six climate regions for the three decadal periods. B) We calculated the population exposed to temperature range by multiplying the proportions from (A) by the corresponding national population from decadal Indian censuses in 1981 (*n* = 683,329,097), 1991 (*n* = 846,427,039), and 2001 (*n* = 1,028,737,436) [46].

Our study has several limitations. First, the temperature data had a coarse spatial resolution of 1 × 1° (roughly 100 km at the equator) [18]. This may hide microclimate effects and lead to biases in either positive or negative directions [47]. But strong correlation between the temperature data and other high-resolution climate data support the usage of the former dataset [18]. Second, the use of daily mean temperature (average of daily minimum and maximum temperatures) is not equivalent to temperature averaged over the whole day. It is possible for the day to be colder than the night in winter. More refined temperature data were not available for analysis. Third, we could not perform two-stage meta-analysis on deaths from specific causes due to their small sample sizes when stratified by climate region. However, meta-analysis on medical deaths showed that between-region heterogeneity arose chiefly for ages 70 years and above. Moreover, the single-stage estimates and the two-stage pooled estimates exhibited strong similarities. Lastly, we did not have daily or weekly air pollution data or other environmental exposures available to consider as confounders. However, it is likely that the relationship of mortality and temperature is distinct from that with air pollution and other environmental confounders [48].

This is the first study providing nationally representative estimates of mortality due to hot and cold temperatures in India. We demonstrate that a substantial number of deaths were attributable to moderately cold temperature. Currently, public health interventions focus on the adverse health impact of extreme heat [49]. Our results suggest that public health sectors should re-evaluate their intervention efforts and consider expanding their focus to include moderately cold temperature [2]. The public should be educated about the adverse impacts of cool temperatures, particularly as the largest absolute growth has been among populations exposed to moderately cold temperatures. Health practitioners should pay attention to the symptoms of cardiovascular and respiratory diseases under such temperature conditions. Lastly, similar large-scale and nationally representative studies in other LMICs are needed to understand the temperature–mortality associations in these countries.

## Supporting information

S1 STROBE Checklist (DOCX)

S1 Appendix Additional method details, tables, and figures. (DOCX)

## Abbreviations

CI: confidence interval
DLNM: distributed-lag nonlinear models
eCI: empirical confidence interval
ICD: International Classification of Diseases
IHD: ischaemic heart disease
LMICs: low- and middle-income countries
MDS: Million Death Study
MMT: minimum mortality temperature
OR: odds ratio
RGI: Registrar General of India
SRS: Sample Registration System
