# Supplementary material for: Mortality attributable to hot and cold ambient temperatures in India: a nationally representative case-crossover study
Source: PLoS Med. 2018 Jul 24;15(7):e1002619. doi: 10.1371/journal.pmed.1002619 (PMC6057641; doi:10.1371/journal.pmed.1002619)
Supplement: S1 STROBE Checklist — (DOCX) [file pmed.1002619.s001.docx]

STROBE Statement—Checklist of items that should be included in reports of ***cross-sectional studies***

| **Item** | Item No | Recommendation | Section, page number, paragraph, excerpts, other comments |
| --- | --- | --- | --- |
| **Title and abstract** | 1 | (*a*) Indicate the study’s design with a commonly used term in the title or the abstract | Title, page 1 |
|  |  | (*b*) Provide in the abstract an informative and balanced summary of what was done and what was found | Abstract, page 2 |
| Introduction | | |  |
| Background/rationale | 2 | Explain the scientific background and rationale for the investigation being reported | Introduction, paragraph 1-2. Excerpt: “In India, the limited evidence on temperature and health risks has focused mostly on the effects of heat waves, and has mostly been local.” |
| Objectives | 3 | State specific objectives, including any prespecified hypotheses | Introduction, paragraph 3. Excerpt on objective: “we quantify heat and cold effects on all medical causes of death for all ages as well as on stroke, ischaemic heart disease, and respiratory diseases among adults aged 30-69 in India.” |
| Methods | | |  |
| Study design | 4 | Present key elements of study design early in the paper | Methods, paragraph 3-4. |
| Setting | 5 | Describe the setting, locations, and relevant dates, including periods of recruitment, exposure, follow-up, and data collection | Methods, paragraph 1-2. |
| Participants | 6 | (*a*) Give the eligibility criteria, and the sources and methods of selection of participants | Methods, paragraph 5-6. Cases were deaths of specific mortality causes (all medical, stroke, ischaemic heart disease, and respiratory diseases). |
| Variables | 7 | Clearly define all outcomes, exposures, predictors, potential confounders, and effect modifiers. Give diagnostic criteria, if applicable | Methods, paragraph 2-5. Excerpts: “We used daily mean temperature as the main exposure”, “This case-crossover model has the benefit of controlling for all time-invariant confounders.”, “We applied a two-stage approach to examine temperature associations with all known medical causes of deaths… by age group (ages 0-29 years, 30-69 years, 70 years and above).” |
| Data sources/ measurement | 8* | For each variable of interest, give sources of data and details of methods of assessment (measurement). Describe comparability of assessment methods if there is more than one group | Methods, paragraph 2 |
| Bias | 9 | Describe any efforts to address potential sources of bias | Methods, paragraph 3. Excerpt: “Matching control days by month and day of the week avoids bias from systematic or slowly-evolving temporal confounders such as day-of-week effects, seasonality, and time trends.” |
| Study size | 10 | Explain how the study size was arrived at | Methods, paragraph 1-2, 5-6. Excerpts: “Address information from these death records permit geocoding of 565282 deaths from 2001-2013”, “We excluded deaths without valid death dates (about 2% of all deaths, n=9557).”, “…excluded the northwestern regions with low death counts (n=5917).” Also see Table 1 in S1 Appendix. |
| Quantitative variables | 11 | Explain how quantitative variables were handled in the analyses. If applicable, describe which groupings were chosen and why | Methods, paragraph 2 |
| Statistical methods | 12 | (*a*) Describe all statistical methods, including those used to control for confounding | Methods, paragraph 3-4 |
|  |  | (*b*) Describe any methods used to examine subgroups and interactions | Methods, paragraph 5 & 7 |
|  |  | (*c*) Explain how missing data were addressed | Methods, paragraph 2. Excerpt: “We excluded deaths without valid death dates (about 2% of all deaths, n=9557).” |
|  |  | (*d*) If applicable, describe analytical methods taking account of sampling strategy | Not applicable |
|  |  | (*e*) Describe any sensitivity analyses | Methods, paragraph 9 |
| Results | | |  |
| Participants | 13* | (a) Report numbers of individuals at each stage of study—eg numbers potentially eligible, examined for eligibility, confirmed eligible, included in the study, completing follow-up, and analysed | Table 1 in S1 Appendix |
|  |  | (b) Give reasons for non-participation at each stage | Table 1 in S1 Appendix |
|  |  | (c) Consider use of a flow diagram | Table is a better option than flow diagram given the number of models examined |
| Descriptive data | 14* | (a) Give characteristics of study participants (eg demographic, clinical, social) and information on exposures and potential confounders | Details are already described in the method section. |
|  |  | (b) Indicate number of participants with missing data for each variable of interest | Details are described in the method section and in Table 1 in S1 Appendix |
| Outcome data | 15* | Report numbers of outcome events or summary measures | Fig 1; Table 1 in S1 Appendix |
| Main results | 16 | (*a*) Give unadjusted estimates and, if applicable, confounder-adjusted estimates and their precision (eg, 95% confidence interval). Make clear which confounders were adjusted for and why they were included | Not applicable. Time-invariant confounders were controlled for by study design. Air pollution data were unavailable for analysis. |
|  |  | (*b*) Report category boundaries when continuous variables were categorized | Category boundaries for temperature ranges can be found in the Methods, paragraph 7 and the column headings of Table 1 & 2 |
|  |  | (*c*) If relevant, consider translating estimates of relative risk into absolute risk for a meaningful time period | We calculated absolute number of temperature-attributable deaths (Table 2) using the attributable risks fractions (Table 1). |
| Other analyses | 17 | Report other analyses done—eg analyses of subgroups and interactions, and sensitivity analyses | Results, paragraph 5 |
| Discussion | | |  |
| Key results | 18 | Summarise key results with reference to study objectives | Discussion, paragraph 1-2. Excerpts: “We demonstrate that cold temperatures contribute to higher attributable risks than hot temperatures in India”, “We identified differences in the temperature-mortality associations between stroke, ischaemic heart disease, and respiratory diseases.” |
| Limitations | 19 | Discuss limitations of the study, taking into account sources of potential bias or imprecision. Discuss both direction and magnitude of any potential bias | Discussion, paragraph 7. Excerpt: “the temperature data had a coarse spatial resolution…lead to biases in either positive or negative directions.” |
| Interpretation | 20 | Give a cautious overall interpretation of results considering objectives, limitations, multiplicity of analyses, results from similar studies, and other relevant evidence | Discussion, paragraph 1,4,5 |
| Generalisability | 21 | Discuss the generalisability (external validity) of the study results | Not applicable. Study is only meant to provide estimates for India. Further research is needed in other LMIC countries. |
| Other information | | |  |
| Funding | 22 | Give the source of funding and the role of the funders for the present study and, if applicable, for the original study on which the present article is based | Provided in online submission |

*Give information separately for exposed and unexposed groups.

**Note:** An Explanation and Elaboration article discusses each checklist item and gives methodological background and published examples of transparent reporting. The STROBE checklist is best used in conjunction with this article (freely available on the Web sites of PLoS Medicine at http://www.plosmedicine.org/, Annals of Internal Medicine at http://www.annals.org/, and Epidemiology at http://www.epidem.com/). Information on the STROBE Initiative is available at www.strobe-statement.org.
