# Supplementary material for: Mortality attributable to hot and cold ambient temperatures in India: a nationally representative case-crossover study
Source: PLoS Med. 2018 Jul 24;15(7):e1002619. doi: 10.1371/journal.pmed.1002619 (PMC6057641; doi:10.1371/journal.pmed.1002619)
Supplement: S1 Appendix — (DOCX) [file pmed.1002619.s002.docx]

# S1 Appendix: Mortality attributable to hot and cold ambient temperatures in India: a nationally representative case-crossover study

| **S1 contents** | **Page** |
| --- | --- |
| **A Methods: parameters for spline functions in main models and sensitivity analyses**  **B Methods: calculations of temperature-attributable number of deaths**  **C Results: additional tables** | 2  2  3-5 |
| **D Results: additional figures** | 6-22 |
|  |  |

**A. Parameters for spline functions in main models and sensitivity analyses**

We modeled the temperature–mortality association with a quadratic B-spline. We placed three internal knots at absolute temperatures that corresponded to the 10th, 75th, and 90th percentiles of Indian temperature distribution [1]. Indian temperature distribution used daily mean temperatures of the temperature grids with Sampling Registration System units. We modeled the lag**–**mortality relationship with a natural cubic B-spline with an intercept. We placed three internal knots at equally-spaced values in the log scale, using a lag period of up to 21 days [1].

In sensitivity analyses, we assessed model results using different knot placements on the quadratic B-spline for the temperature–mortality associations of both two-stage meta-analyses for medical deaths and cause-specific models. We examined the following: 1) five and nine internal knots placed at equally-spaced percentile values and 2) internal knots placed at every 10 °C (12.5 °C, 22.5 °C, 32.5 °C).

**B. Calculations of temperature-attributable number of deaths**

We calculated the temperature-attributable number of deaths using the following equation:

$${AN}_{i,t}={AF}_{i,t}\times(\sum_{x}^{n} {Pr}_{i,x} \times{UN}_{x})$$

Where *i* represents mortality cause and *t* is temperature range; *x* represents five-year age group and *n* is the total number of five-year age groups. *AN* is the attributable number of deaths and *AF* is the attributable risk fraction for 2001–2013; *Pr* is proportionate mortality, that is, the proportion of cause-specific deaths among all deaths from 2001–202013; *UN* is the United Nations’ estimates of total deaths in India for 2015 [2].

**C. Additional tables**

**S1 Table A. International Classification of Diseases (ICD)-10 codes and number of deaths by mortality cause and age group**

| **Mortality cause** | **ICD-10 codes** | **Age group** | **Number of deaths** | | | | |
| --- | --- | --- | --- | --- | --- | --- | --- |
|  |  |  | **All MDS data** | **Geocoded** | **Geocoded with death dates** | **Belonged to six climate regions*** | |
|  |  |  |  |  |  | **All** | **With missing temperature data up to 21 lag days** |
| All | A00-Z99 | All ages | 591121 | 565282 | 555725 | 546360 | Not analyzed |
| Medical† | A00-Q99, R04-06, R55, R84, R91, R96, X30-31 | All ages | 440445 | 426259 | 418990 | 411613 | 648 |
|  |  | 0-29 | 117616 | 113427 | 111039 | 109291 | 193 |
|  |  | 30-69 | 196023 | 189950 | 186930 | 183388 | 327 |
|  |  | 70+ | 126724 | 122805 | 120950 | 118863 | 128 |
| Stroke | G45-46, I60-67, I69, G81-83 | 30-69 | 21209 | 20508 | 20149 | 19753 | 42 |
|  |  | 70+ | 19381 | 18753 | 18454 | 18166 | 25 |
| Ischaemic Heart Diseases | I20-25, I46, R55, R96 | 30-69 | 41989 | 41274 | 40729 | 40003 | 37 |
|  |  | 70+ | 21902 | 21505 | 21231 | 20905 | 11 |
| Respiratory diseases | H65-68, H70-71, J00-64, J66-99, P23, R04-06, R84, R91 | 30-69 | 25090 | 24583 | 24238 | 23595 | 31 |
|  |  | 70+ | 29756 | 29207 | 28797 | 28099 | 28 |

* Excluded deaths from the northern regions and those that could not be assigned a climate region.

† Death counts by age group do not add up to death counts for all ages due to missing information on age for small number of deaths (82 for all MDS data, 77 for geocoded deaths, 71 for geocoded deaths with death dates, and 71 for deaths belonged to the six climate regions).

**S1 Table B. Proportion of days within temperature ranges in India by Köppen–Geiger climate region and decadal period.**

| **Period & Region** | **Proportion of days within temperature range in India** | | | |
| --- | --- | --- | --- | --- |
|  | **Extremely cold (-0.3 to 13.8 °C)** | **Moderately cold (13.8 to 30.0 °C)** | **Moderately hot (30.0 to 34.2 °C)** | **Extremely hot (34.2 to 40.2 °C)** |
| **Equatorial monsoon (Am)** |  |  |  |  |
| 1981–1990 | 0.27% | 97.55% | 2.18% | < 0.00% |
| 1991–2000 | 0.32% | 97.53% | 2.15% | < 0.00% |
| 2001–2010 | 0.17% | 96.87% | 2.96% | < 0.00% |
|  |  |  |  |  |
| **Equatorial savannah with dry winter (Aw)** |  |  |  |  |
| 1981–1990 | 0.14% | 83.16% | 14.90% | 1.80% |
| 1991–2000 | 0.17% | 83.25% | 14.56% | 2.02% |
| 2001–2010 | 0.11% | 81.75% | 15.90% | 2.24% |
|  |  |  |  |  |
| **Hot steppe climate (Bsh)** |  |  |  |  |
| 1981–1990 | 2.65% | 77.71% | 16.85% | 2.80% |
| 1991–2000 | 2.64% | 77.84% | 16.55% | 2.96% |
| 2001–2010 | 2.57% | 75.84% | 18.48% | 3.12% |
|  |  |  |  |  |
| **Hot desert climate (Bwh)** |  |  |  |  |
| 1981–1990 | 3.97% | 68.06% | 23.76% | 4.21% |
| 1991–2000 | 3.88% | 68.86% | 22.98% | 4.28% |
| 2001–2010 | 3.93% | 65.60% | 25.85% | 4.63% |
|  |  |  |  |  |
| **Warm temperate climate with hot summer and dry winter (Cwa)** |  |  |  |  |
| 1981–1990 | 4.58% | 82.18% | 11.14% | 2.10% |
| 1991–2000 | 4.65% | 81.70% | 11.42% | 2.23% |
| 2001–2010 | 4.32% | 80.30% | 12.92% | 2.46% |
|  |  |  |  |  |
| **Warm temperate climate with warm summer and dry winter (Cwb)** |  |  |  |  |
| 1981–1990 | 8.84% | 87.78% | 3.35% | 0.03% |
| 1991–2000 | 9.47% | 87.22% | 3.25% | 0.06% |
| 2001–2010 | 8.10% | 87.88% | 4.00% | 0.03% |

Note: Proportions were calculated by combining daily mean temperatures from temperature grids of the six climate regions.

| **S1 Table C. Attributable risk fractions for India compared to other countries** | | | | |
| --- | --- | --- | --- | --- |
|  |  |  |  |  |
|  | **Minimum mortality percentile** | **Total**  **% (95% eCI)** | **Cold**  **% (95% eCI)** | **Hot**  **% (95% eCI)** |
| **Indian pooled estimates at all ages** | 82rd | 7.32% (2.20 to 11.98) | 6.83% (1.36 to 11.57) | 0.49% (0.06 to 0.91) |
| **International study** |  |  |  |  |
| Australia | 83th | 6.96% (4.27 to 9.51) | 6.50% (3.91 to 8.94) | 0.45% (0.20 to 0.70) |
| Brazil | 60th | 3.53% (3.00 to 4.01) | 2.83% (2.34 to 3.30) | 0.70% (0.45 to 0.93) |
| Canada | 81st | 5.00% (3.83 to 6.07) | 4.46% (3.39 to 5.48) | 0.54% (0.39 to 0.66) |
| China | 83rd | 11.00% (9.29 to 12.47) | 10.36% (8.72 to 11.77) | 0.64% (0.47 to 0.79) |
| Italy | 79th | 10.97% (8.03 to 13.43) | 9.35% (6.59 to 11.72) | 1.62% (1.24 to 1.98) |
| Japan | 86th | 10.12% (9.61 to 10.56) | 9.81% (9.32 to 10.22) | 0.32% (0.27 to 0.36) |
| South Korea | 89th | 7.24% (4.45 to 9.73) | 6.93% (4.12 to 9.44) | 0.31% (0.15 to 0.45) |
| Spain | 78th | 6.52% (5.82 to 7.16) | 5.46% (4.79 to 6.07) | 1.06% (0.96 to 1.16) |
| Sweden | 93rd | 3.87% (–6.20 to 12.93) | 3.69% (–6.31 to 12.61) | 0.18% (–0.47 to 0.65) |
| Taiwan | 62nd | 4.75% (3.26 to 6.06) | 3.89% (2.50 to 5.31) | 0.86% (0.12 to 1.50) |
| Thailand | 60th | 3.37% (3.06 to 3.63) | 2.61% (2.31 to 2.88) | 0.76% (0.65 to 0.86) |
| UK | 90th | 8.78% (8.00 to 9.54) | 8.48% (7.72 to 9.25) | 0.30% (0.25 to 0.36) |
| USA | 84th | 5.86% (5.50 to 6.17) | 5.51% (5.17 to 5.82) | 0.35% (0.30 to 0.39) |
| Total | 81st | 7.71% (7.43 to 7.91) | 7.29% (7.02 to 7.49) | 0.42% (0.39 to 0.44) |

Note: Attributable mortality computed as total and as separate components for cold and heat. Estimates for international study were adapted from Gasparrini et al. 2015[1]. eCI, empirical confidence interval

**D. Additional figures**

Abbreviations in figures and footnotes:

CI, confidence interval

MMT, minimum mortality temperature

OR, odds ratio


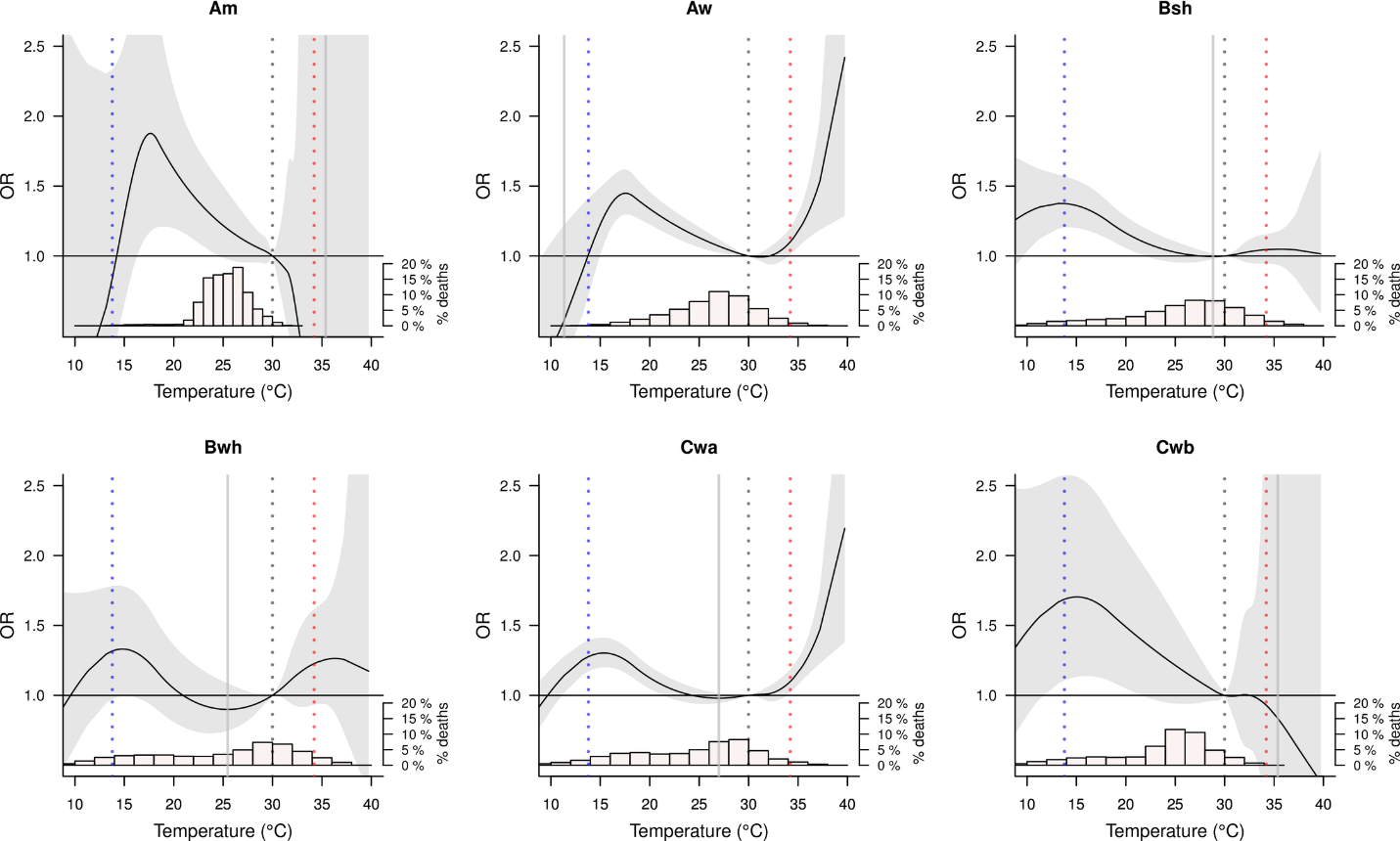


**S1 Fig A.** **Overall cumulative temperature**–**mortality associations for first-stage estimates at all ages.** Black curves show the temperature–mortality associations with 95% CIs (shaded grey). Histograms show the proportion of deaths by daily mean temperature of the death date. Vertical grey solid lines and dotted lines represent the region-specific MMTs and pooled estimate MMT, respectively. All curves are centred on the pooled estimate MMT. Vertical blue and red dotted lines indicate the 2.5th and 97.5th percentiles of Indian temperature distribution. Graphs are restricted to 10 °C**–**40 °C due to wide CIs for extremely cold temperature.


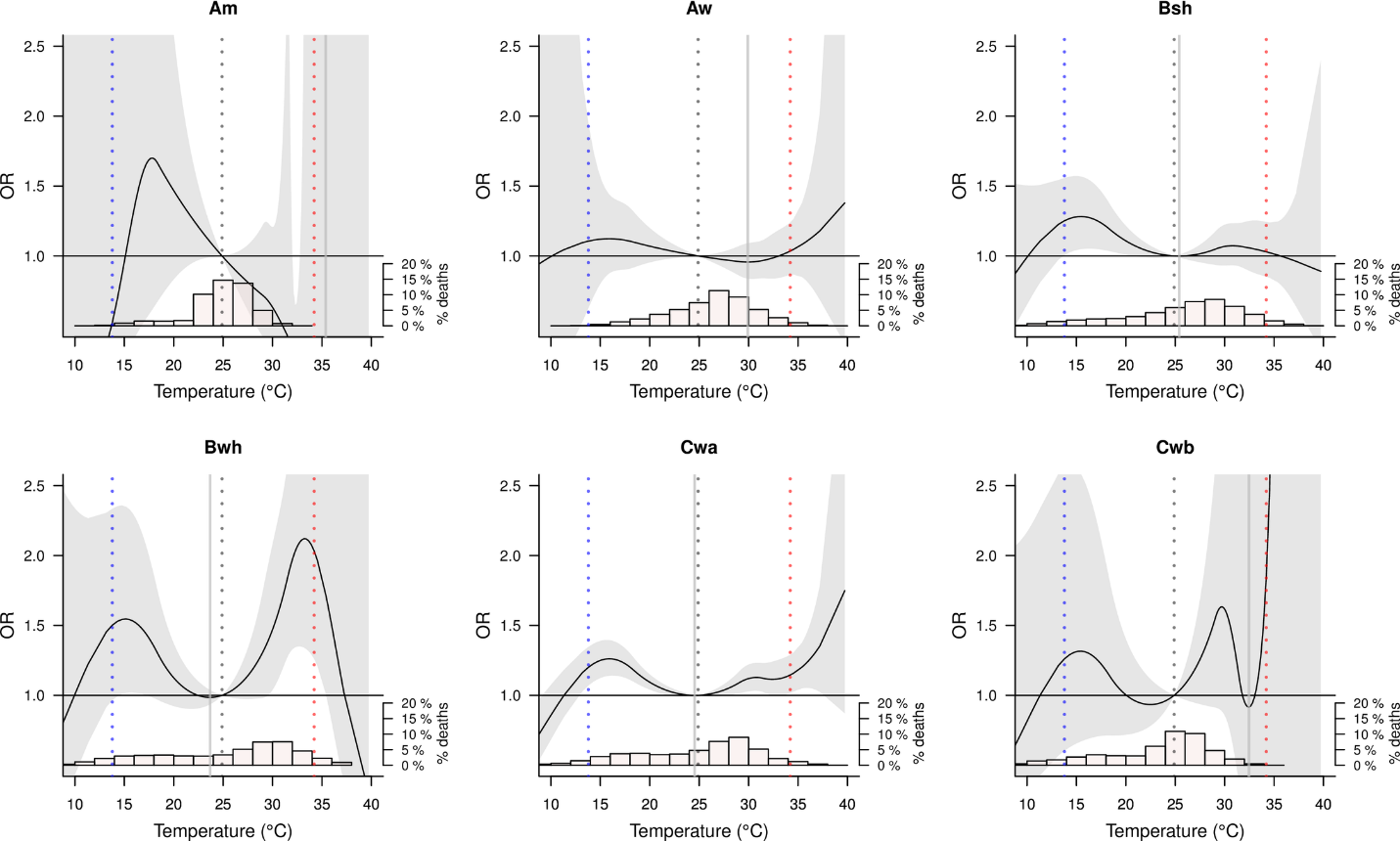


**S1 Fig B.** **Overall cumulative temperature**–**mortality associations for first-stage estimates at ages 0–29 years.** Black curves show the temperature–mortality associations with 95% CIs (shaded grey). Histograms show the proportion of deaths by daily mean temperature of the death date. Vertical grey solid lines and dotted lines represent the region-specific MMTs and pooled estimate MMT, respectively. All curves are centred on the pooled estimate MMT. Vertical blue and red dotted lines indicate the 2.5th and 97.5th percentiles of Indian temperature distribution. Graphs are restricted to 10 °C **–**40 °C due to wide CIs for extremely cold temperature.


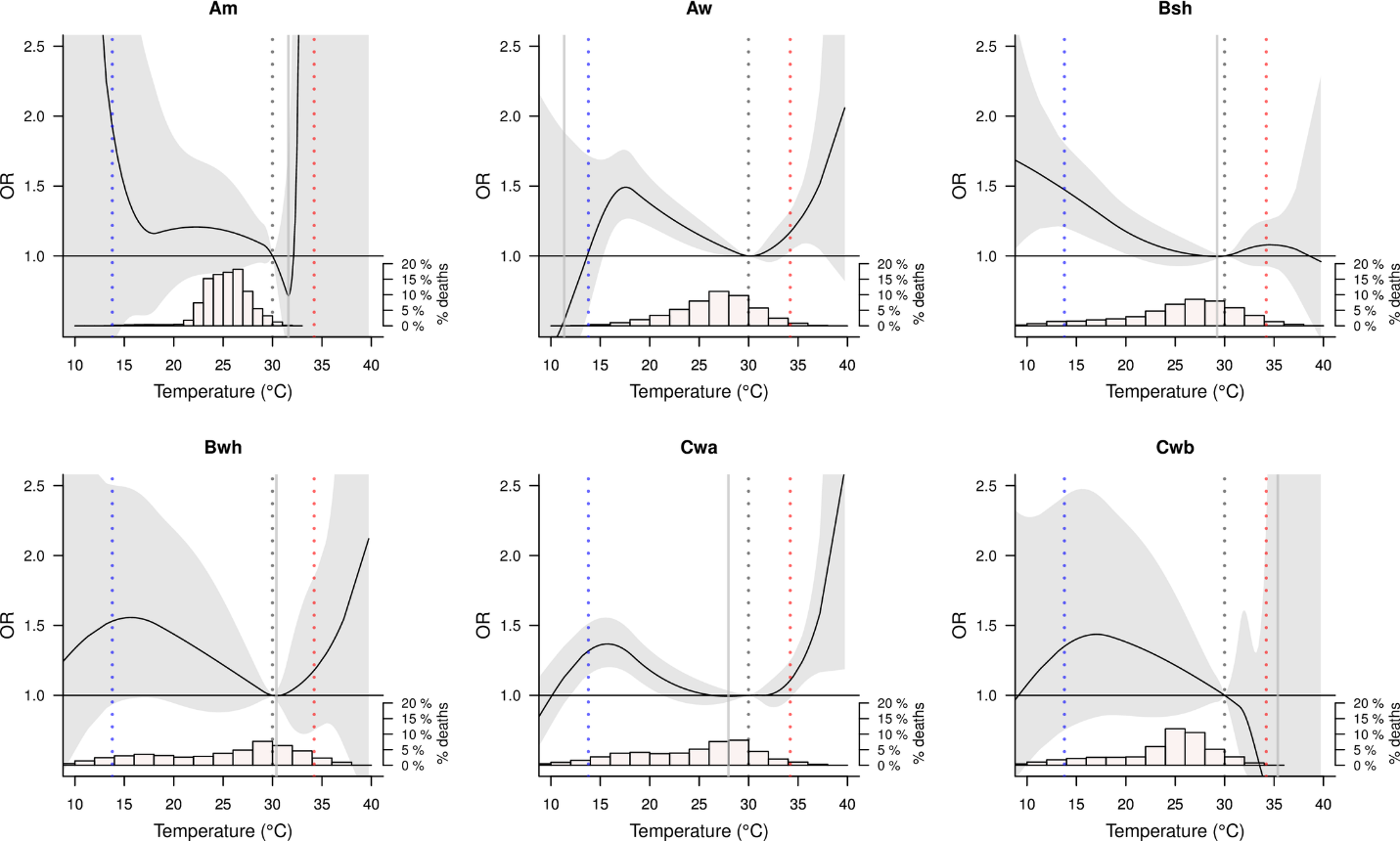


**S1 Fig C.** **Overall cumulative temperature**–**mortality associations for first-stage estimates at ages 30–69 years.** Black curves show the temperature–mortality associations with 95% CIs (shaded grey). Histograms show the proportion of deaths by daily mean temperature of the death date. Vertical grey solid lines and dotted lines represent the region-specific MMTs and pooled estimate MMT, respectively. All curves are centred on the pooled estimate MMT. Vertical blue and red dotted lines indicate the 2.5th and 97.5th percentiles of Indian temperature distribution. Graphs are restricted to 10 °C**–**40 °C due to wide CIs for extremely cold temperature.


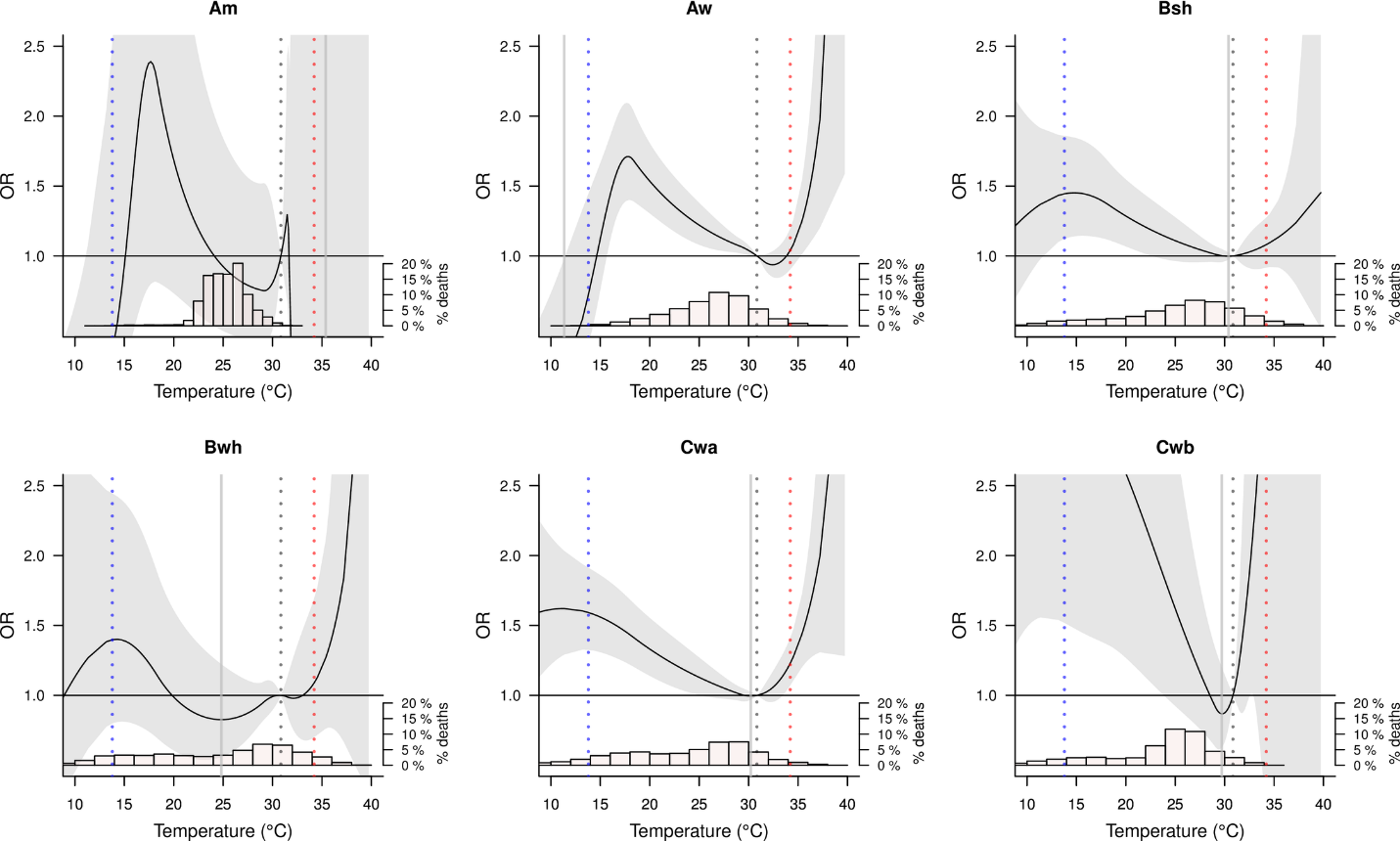


**S1 Fig D.** **Overall cumulative temperature**–**mortality associations for first-stage estimates at ages 70 years and above.** Black curves show the temperature–mortality associations with 95% CIs (shaded grey). Histograms show the proportion of deaths by daily mean temperature of the death date. Vertical grey solid lines and dotted lines represent the region-specific MMTs and pooled estimate MMT, respectively. All curves are centred on the pooled estimate MMT. Vertical blue and red dotted lines indicate the 2.5th and 97.5th percentiles of Indian temperature distribution. Graphs are restricted to 10 °C**–**40 °C due to wide CIs for extremely cold temperature.


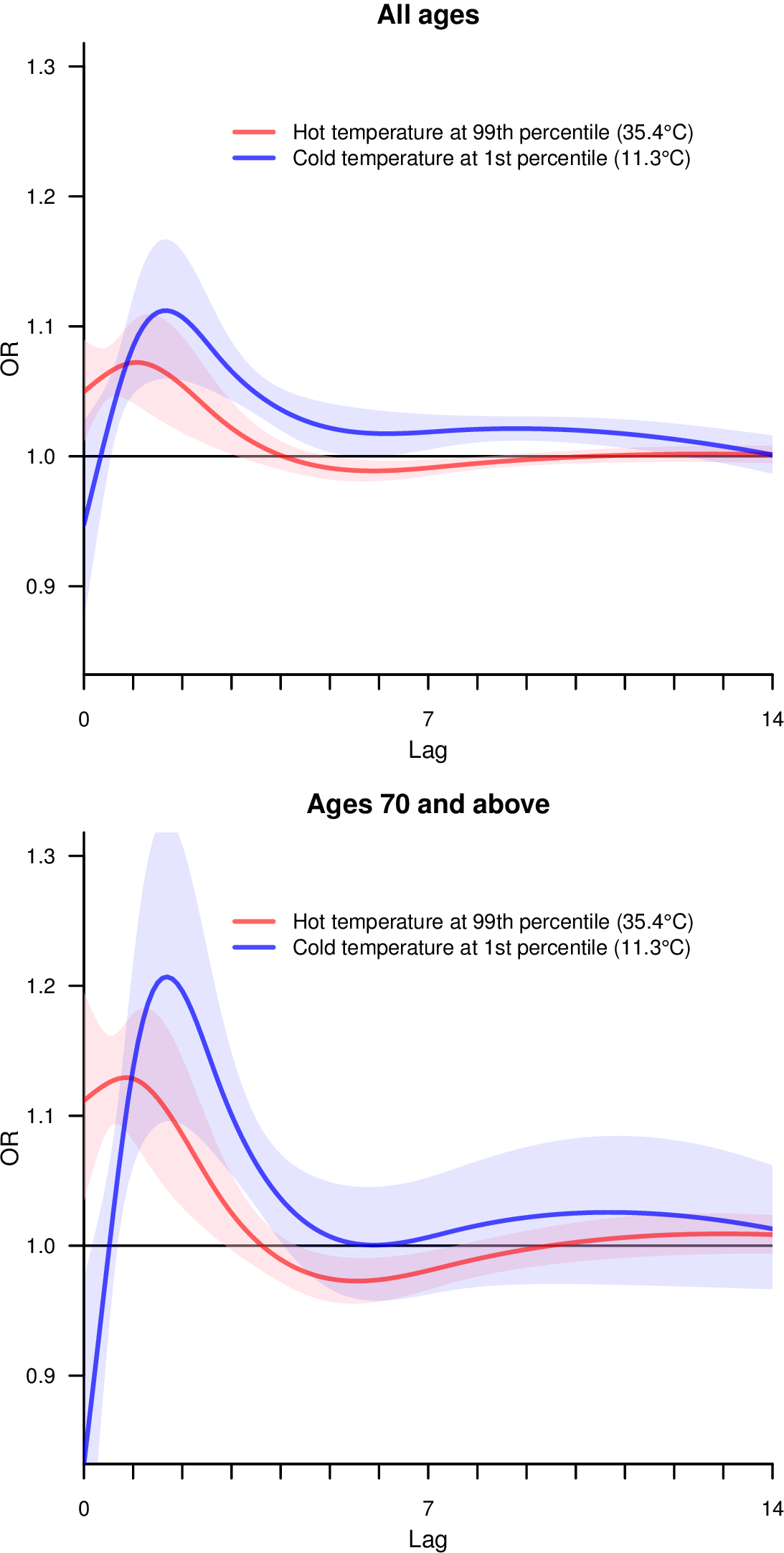


**S1 Fig E. Lag–mortality associations from pooled estimates for medical deaths at all ages and ages 70 years and above.** Solid curves represent lag**–**mortality associations and shadings represent 95% CIs (red: hot temperature at 99th percentile, blue: cold temperature at 1st percentile). Graphs are restricted to 0**–**14 lag days due to non-significant or negative ORs at 15**–**21 lag days.

***
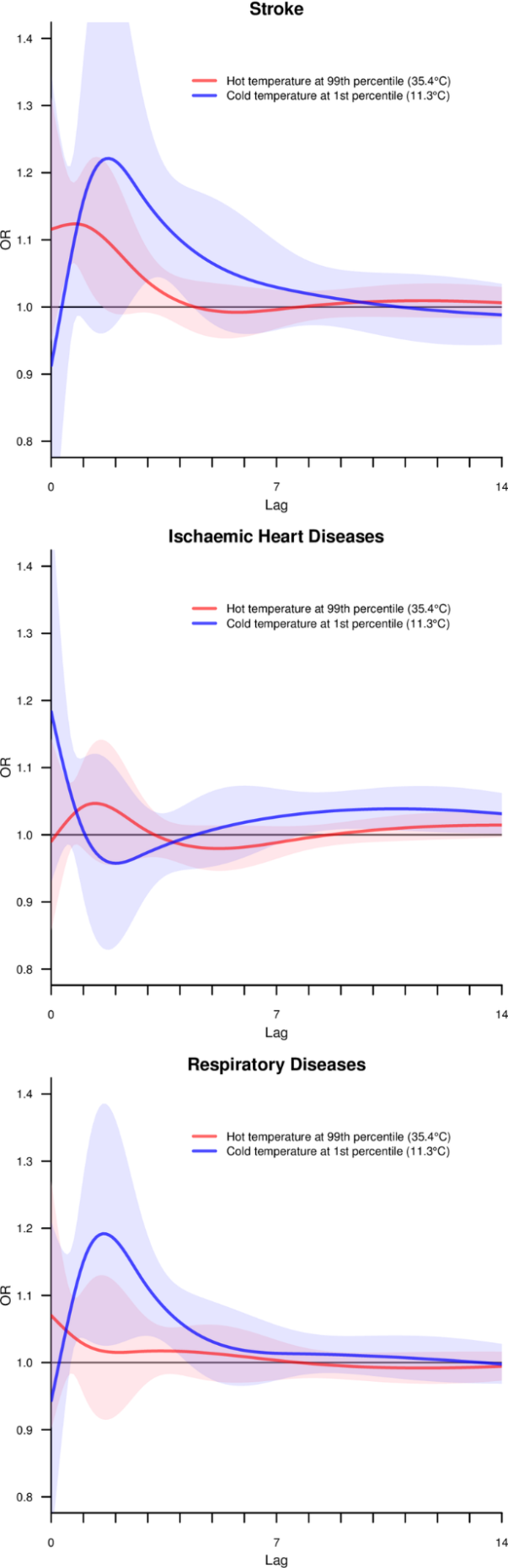
***

**S1 Fig F.** **Lag–mortality associations for specific mortality causes at ages 30–69 years.** Solid curves represent lag**–**mortality associations and shadings represent 95% CIs (red: hot temperature at 99th percentile, blue: cold temperature at 1st percentile). Graphs are restricted to 0**–**14 lag days due to non-significant or negative ORs at 15**–**21 lag days.


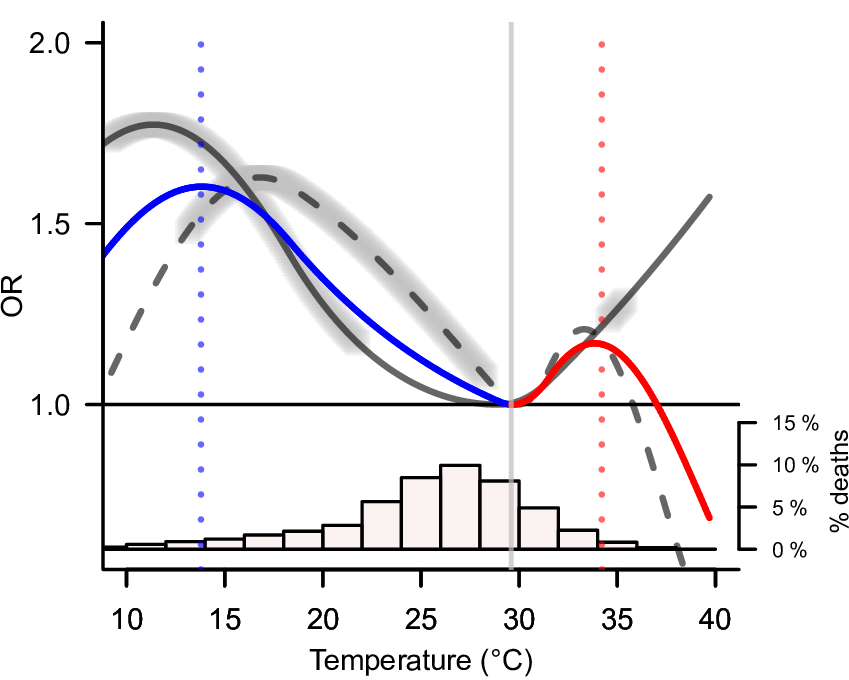


**S1 Fig G. Overall cumulative temperature**–**mortality associations for ischaemic heart disease at ages 30–69 years by previous history of vascular disease.** Solid curve in blue (estimates below MMT) and red (estimates above MMT) shows the original estimates regardless of medical history. Solid and dashed curves in dark grey show estimates for deaths with and without previous history of vascular disease**,** respectively; grey shadings indicate temperature ranges with statistical significance at 95% CIs for the solid and dashed curves. Histogram shows the proportion of deaths from ischaemic heart disease regardless of medical history by daily mean temperature of the death date. Vertical grey solid line represents MMT from the original estimates regardless of medical history. Vertical blue and red dotted lines indicate the 2.5th and 97.5th percentiles of Indian temperature distribution. Graphs are restricted to 10 °C**–**40 °C due to wide CIs for extremely cold temperature.


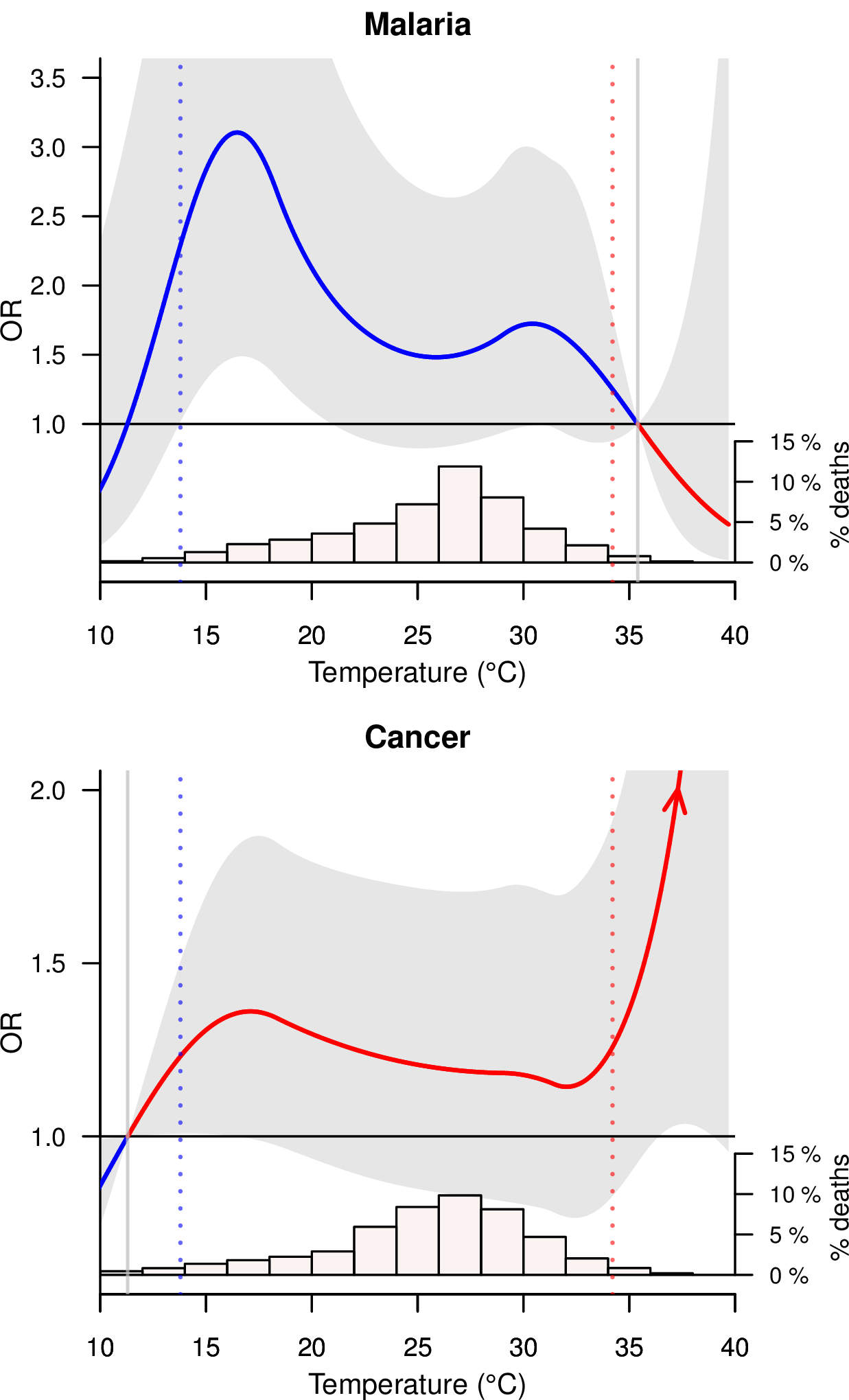


**S1 Fig H.** **Overall cumulative temperature**–**mortality associations for malaria and cancers at ages 30–69 years.** Solid curves show the temperature–mortality associations (blue and red colours for estimates below and above MMTs, respectively) with 95% CIs (shaded grey); red arrow indicates increasing ORs for hot temperatures. Histograms show the proportion of deaths by daily mean temperature of the death date. Vertical grey solid lines represent the model-specific MMTs. Vertical blue and red dotted lines indicate the 2.5th and 97.5th percentiles of Indian temperature distribution. Graphs are restricted to 10 °C**–**40 °C due to wide CIs for extremely cold temperature.

**
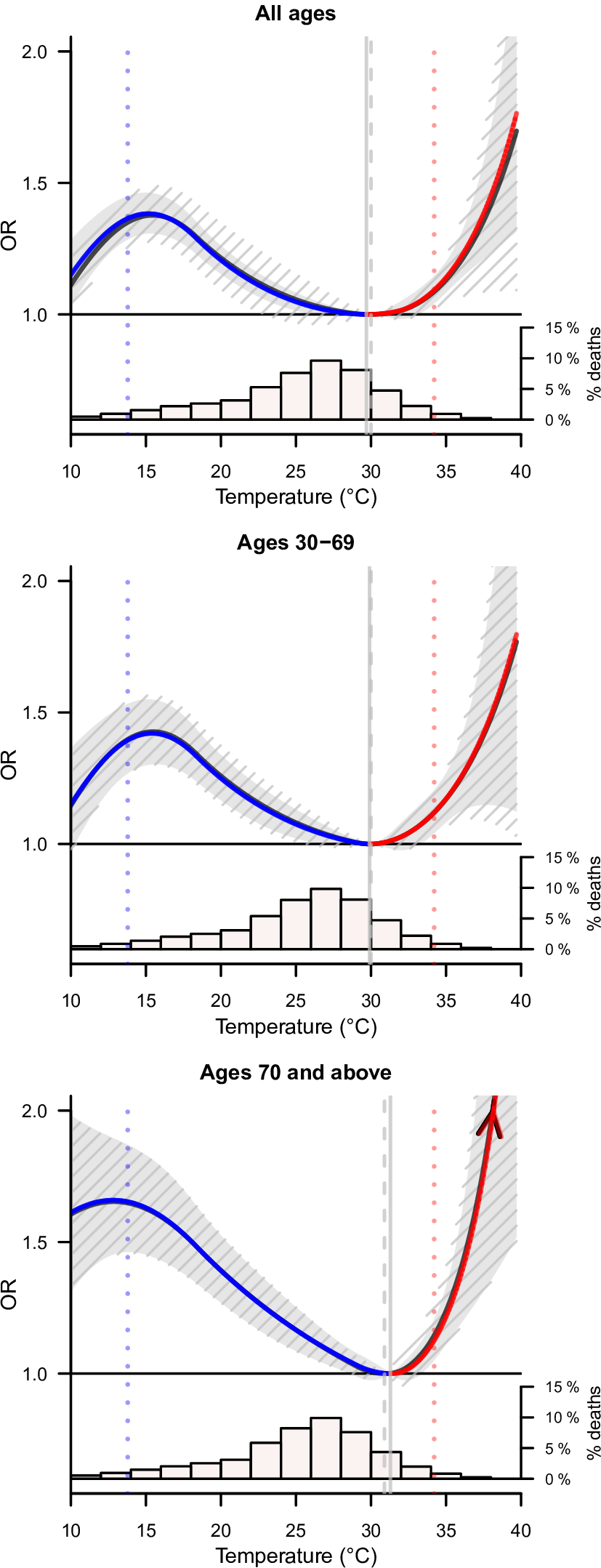
**

**S1 Fig I. Overall cumulative temperature**–**mortality associations of single-stage estimates and two-stage pooled estimates for medical deaths from all ages, ages 30–69 years, and ages 70 years and above.** Solid curves in blue (estimates below MMTs) and red (estimates above MMTs) show single-stage estimates with 95% CIs (grey shadings). Solid curves in black show pooled estimates with 95% CIs (grey hatchings); red and black arrows indicate increasing ORs for hot temperatures for single-stage and pooled estimates, respectively. Histograms show the proportion of deaths by daily mean temperature of the death date. Vertical grey solid lines and grey long-dashed lines represent the MMTs from single-stage and pooled estimates, respectively. Vertical blue and red dotted lines indicate the 2.5th and 97.5th percentiles of Indian temperature distribution. Graphs are restricted to 10 °C**–**40 °C due to wide CIs for extremely cold temperature.

**The following S1 figures (J-O) present results from sensitivity analyses on knot placements for the temperature**–**mortality associations:** **panel A, five knots at equally-spaced percentile values; panel B, nine knots at equally-spaced percentile values; panel C, three internal knots at every 10 °C.** Solid curves show the temperature–mortality associations (blue and red colours for estimates below and above MMTs, respectively) with 95% CIs (shaded grey); red arrows indicate increasing ORs for hot temperatures. Histograms show the proportion of deaths by daily mean temperature of the death date. Vertical grey solid lines represent the model-specific MMTs. Vertical blue and red dotted lines indicate the 2.5th and 97.5th percentiles of Indian temperature distribution. Graphs are restricted to 10 °C**–**40 °C due to wide CIs for extremely cold temperature.


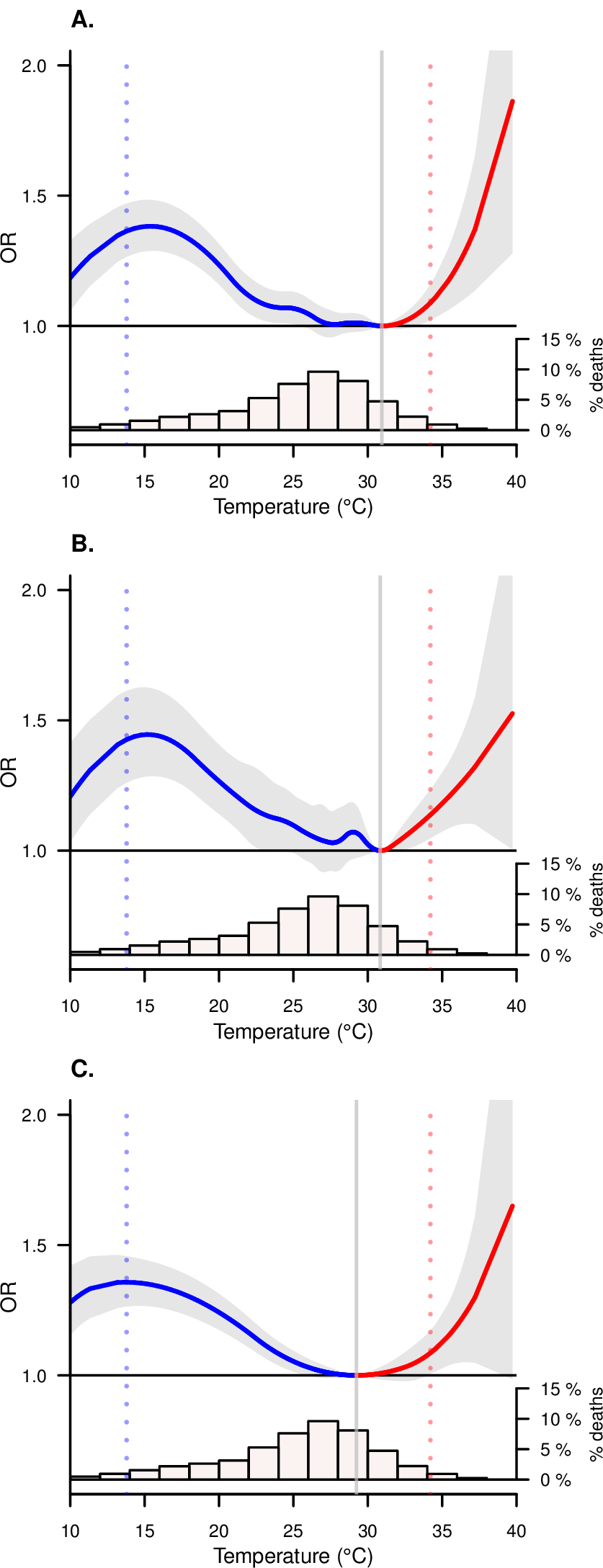


**S1 Fig J. Sensitivity analyses on knot placements for the temperature**–**mortality associations for medical deaths from all ages.**

**
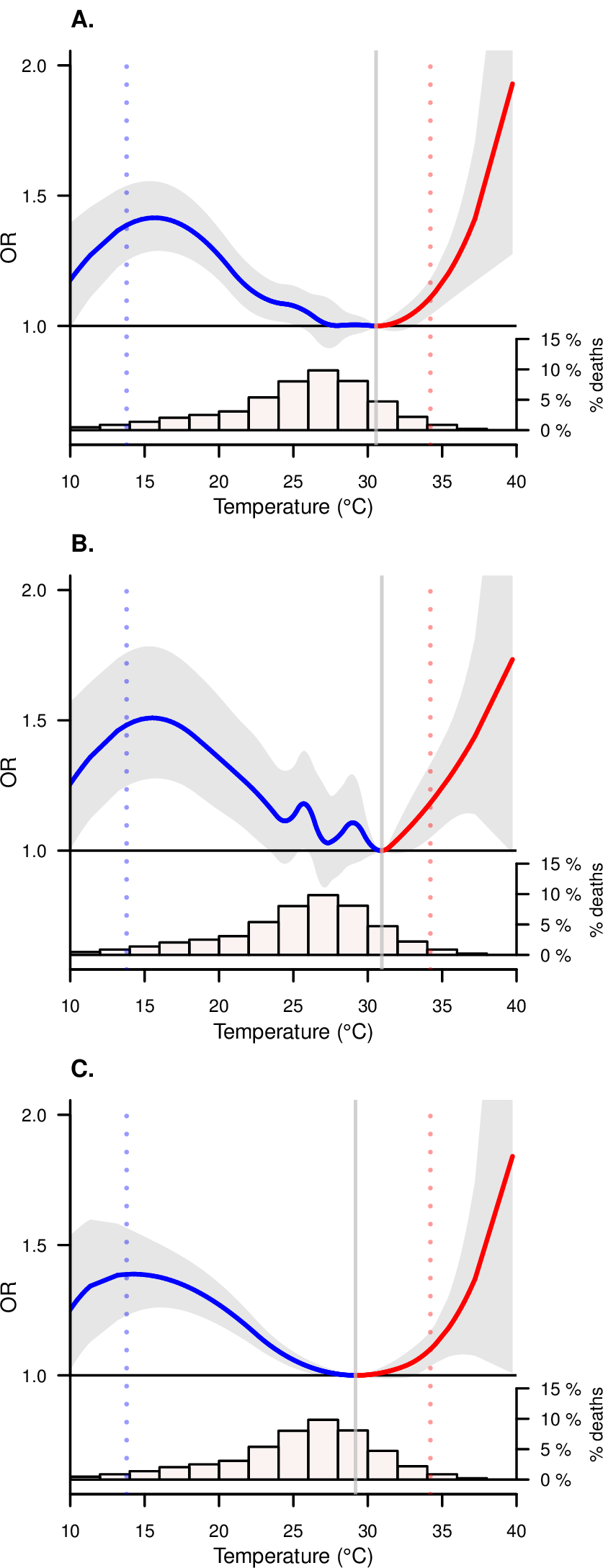
**

**S1 Fig K. Sensitivity analyses on knot placements for the temperature**–**mortality associations for medical deaths from ages 30–69 years.**

**
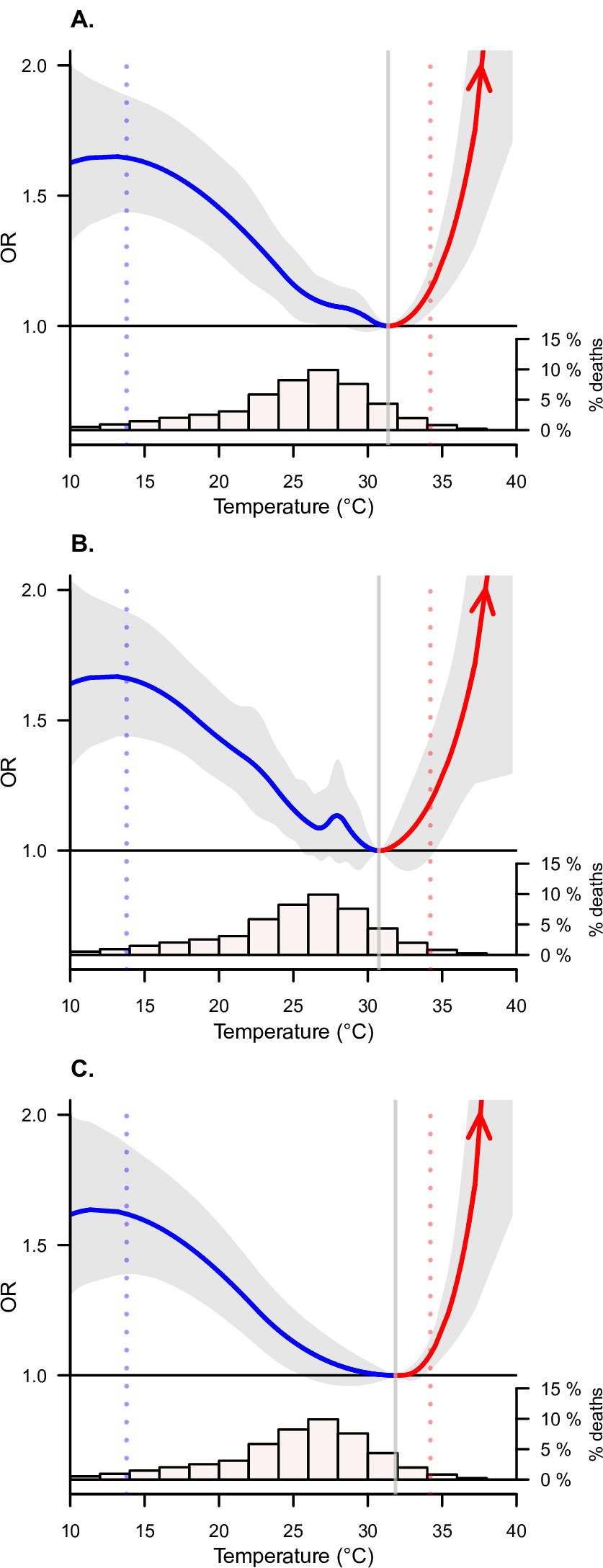
**

**S1 Fig L. Sensitivity analyses on knot placements for the temperature**–**mortality associations for medical deaths from ages 70 years and above.**


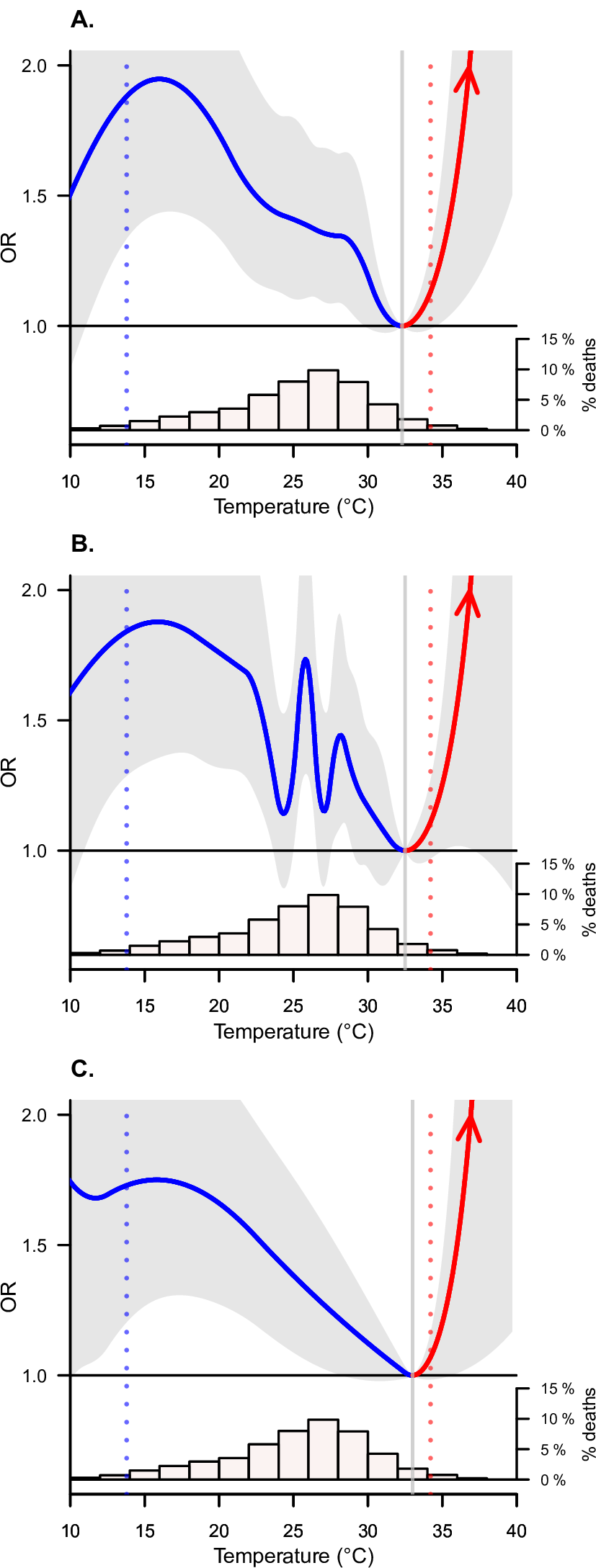


**S1 Fig M. Sensitivity analyses on knot placements for the temperature**–**mortality associations for stroke deaths from ages 30–69 years.**


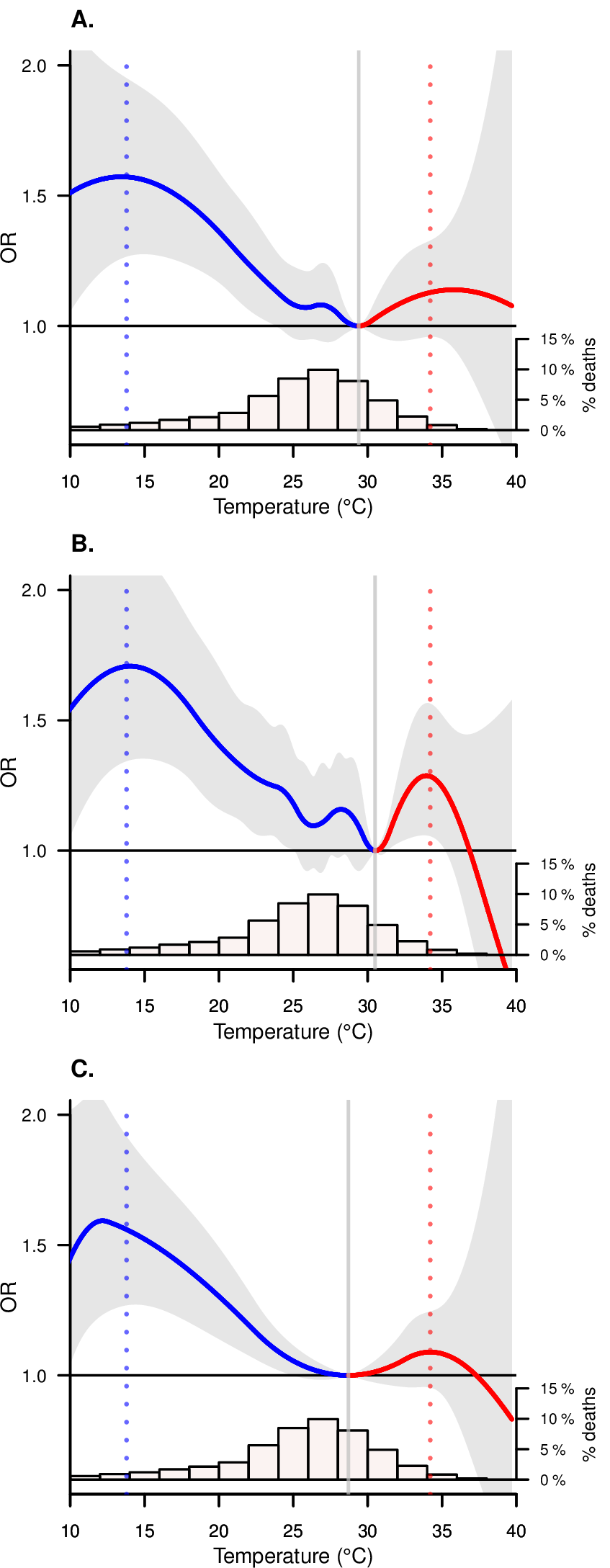


**S1 Fig N. Sensitivity analyses on knot placements for the temperature**–**mortality associations for ischaemic heart disease deaths from ages 30–69 years.**


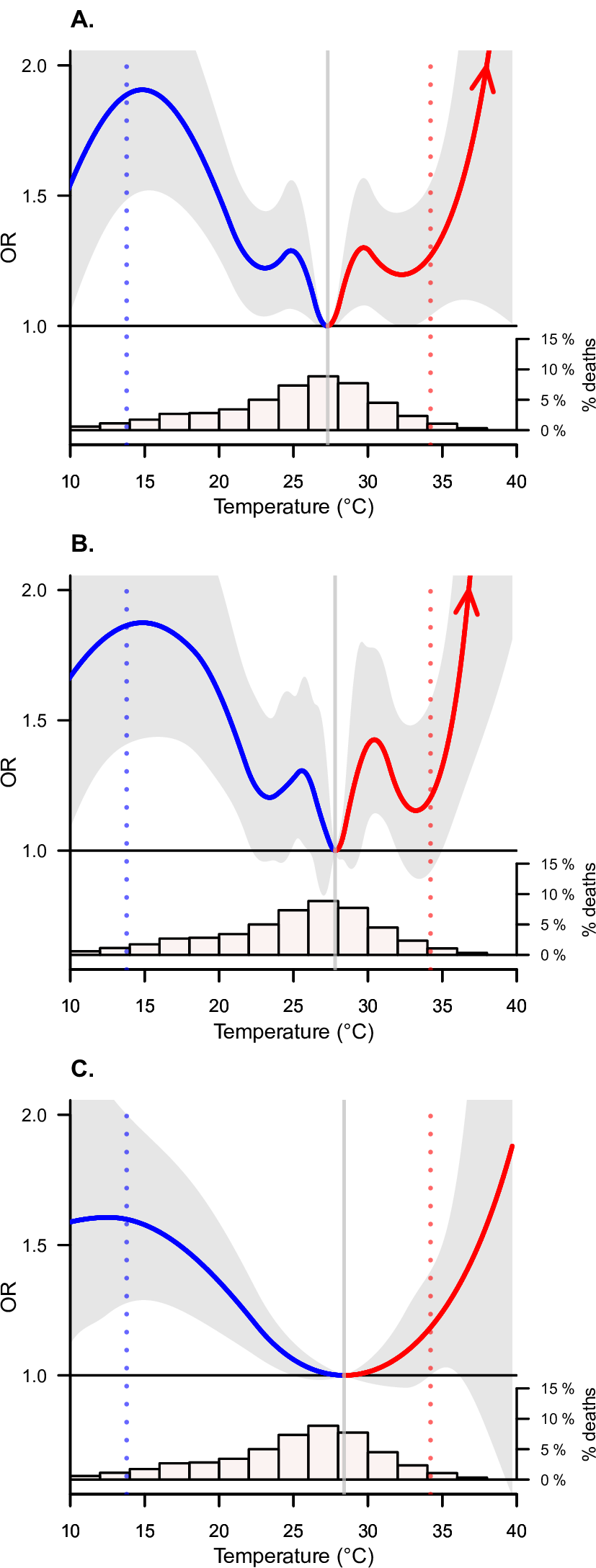


**S1 Fig O. Sensitivity analyses on knot placements for the temperature**–**mortality associations for respiratory diseases deaths from ages 30–69 years.**

**
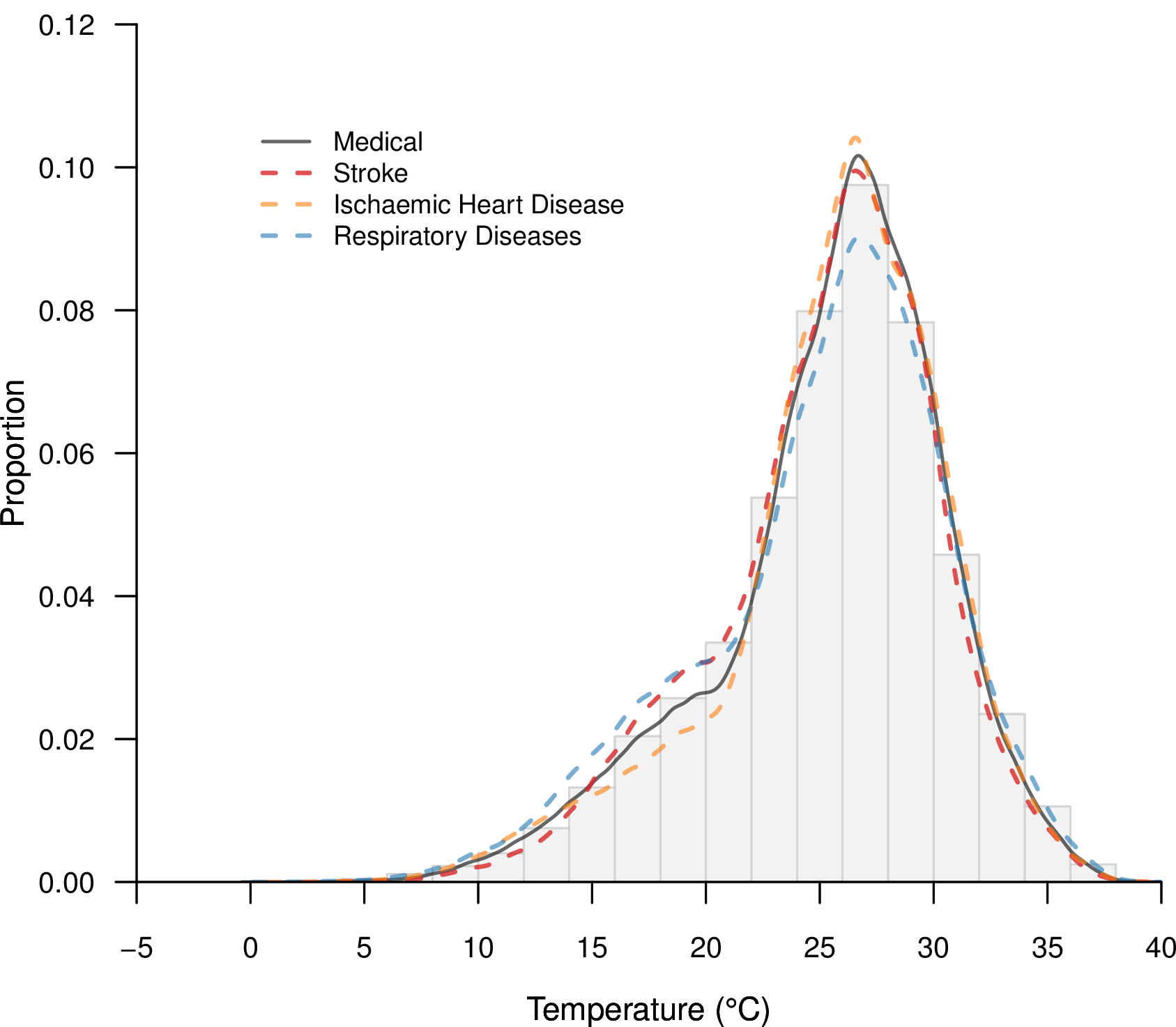
**

**S1 Fig P. Distributions of daily mean temperatures in India.** Histogram represents proportion of daily mean temperatures from temperature grids that belong to the six climate regions in 2001–2013. Dark grey solid curve represents the proportion of medical deaths from ages 30**–**69 years by daily mean temperature of the death date. Red, orange, and blue dashed curves represent the proportion of stroke, ischaemic heart disease, and respiratory diseases deaths, respectively, from ages 30**–**69 years by daily mean temperature of the death date.

**References**

1. Gasparrini, A., et al., *Mortality risk attributable to high and low ambient temperature: a multicountry observational study.* Lancet, 2015. **386**(9991): p. 369-75.

2. United Nations, Department of Economic and Social Affairs, and and Population Division, *The World Population Prospects: 2015 Revision, Key Findings and Advance Tables*. 2015, United Nations: New York.
